# Targeting Chk1 and Wee1 kinases enhances radiosensitivity of 2D and 3D head and neck cancer models to X-rays and low/high-LET protons

**DOI:** 10.1038/s41419-025-07435-0

**Published:** 2025-02-25

**Authors:** Emma Melia, Anne-Sophie Fisch, Ingeborg Tinhofer, Jason L. Parsons

**Affiliations:** 1 Department of Cancer and Genomic Sciences, University of Birmingham, Edgbaston, Birmingham, B15 2TT UK; 2 Department of Radiooncology and Radiotherapy, Translational Radiation Oncology Research Laboratory, Charité – Universitätsmedizin Berlin, corporate member of Freie Universität Berlin and Humboldt Universität zu Berlin, Charitéplatz 1, 10117 Berlin, Germany; 3 School of Physics and Astronomy, University of Birmingham, Edgbaston, Birmingham, B15 2TT UK

**Keywords:** Head and neck cancer, Cell death, Checkpoints

## Abstract

Ionising radiation causes the introduction of DNA damage, more specifically double strand breaks (DSBs) and complex DNA damage (CDD), that induces cancer cell death leading to the therapeutic effect. To combat this, cells activate arrest at the G_2_/M checkpoint to allow for effective DNA damage repair, coordinated by the Chk1 and Wee1 protein kinases. Therefore, Chk1 and Wee1 are considered promising therapeutic targets to enhance the effectiveness of radiotherapy in cancer cell killing. Here, we have analysed the response of head and neck squamous cell carcinoma (HNSCC) cell lines, spheroids and patient-derived organoids to X-rays and proton beam therapy (PBT) in the presence of either a Chk1 (MK-8776) or a Wee1 (MK-1775) inhibitor. We demonstrate that inhibitors of Chk1 or Wee1 can significantly enhance the radiosensitivity of both 2D and 3D models of HNSCC to X-rays and PBT (performed at both low and high ionisation densities), and that this effect is caused through abrogation of the G_2_/M checkpoint causing the persistence of DSBs. Our results therefore suggest that targeting Chk1 and Wee1 kinases in combination with X-rays and PBT could represent a promising therapeutic avenue to enhance the clinical efficacy of HNSCC treatment.

## Introduction

Incidences of head and neck squamous cell carcinoma (HNSCC) are on the rise, currently standing at 890,000 cases and 450,000 deaths per year worldwide [1]. Conventional X-ray radiotherapy is frequently used for the treatment of HNSCC, however patients can suffer from acute and long term adverse side effects, but also many tumours are inherently resistant to the treatment. Proton beam therapy (PBT) is increasingly being used as an alternative form of radiotherapy for HNSCC patients [2], as this is more precision targeted and the radiation dose can be largely confined to the tumour via the Bragg peak. However, there are still major biological and clinical uncertainties with PBT and how this may differ to conventional radiotherapy, largely due to the increases in linear energy transfer (LET) at and around the Bragg peak. Increases in LET lead to enhanced relative biological effectiveness (RBE), somewhat reflected in the RBE of 1.1 for PBT that is used clinically, although this is highly debated [3, 4]. Consequently, further studies are needed to understand the comparative radiobiology of X-rays versus PBT in well characterised HNSCC cell models, but then to identify and utilise targeted drugs/inhibitors against specific cellular pathways that enhance the effectiveness of the radiotherapy treatments and enhance patient survival.

One of the most promising strategies to enhance the effectiveness of radiotherapy is to target the cellular DNA damage response. The major pathways involved in the repair of DNA double strand breaks (DSBs) through non-homologous end-joining (NHEJ) and homologous recombination (HR), and specific enzymes co-ordinating these pathways such as poly(ADP-ribose) polymerase-1 (PARP-1), ataxia telangiectasia mutated (ATM), ataxia telangiectasia and Rad3-related (ATR) and DNA protein kinase (DNA Pk), continue to be investigated as targets for radiosensitisation [5, 6]. Indeed, we have previously demonstrated the effect of targeting these proteins on both 2D monolayers and 3D spheroids models of HNSCC to enhance both X-ray and PBT efficacy [7, 8]. Although this provides a promising avenue, an alternative strategy is to target the cell cycle and to prevent radiation-induced cell cycle arrest which tumour cells use for promoting efficient DNA damage repair, thus driving radiotherapy resistance. Therefore, inhibitors against cell cycle checkpoint kinases, such as Chk1 and Wee1, have been developed and which is given more importance due to the fact that the majority of HNSCC tumours harbour p53 mutations. Consequently, these tumours are reliant on the G_2_/M cell cycle checkpoint driven by Chk1 and Wee1.

It has been previously shown that HNSCC cells have increased X-ray radiosensitivity when combined with the Chk1 inhibitors PF-00477736 [9] or MK-8776 [10]. More recently, it was observed that pre-treatment with AZD7762 significantly increased radiosensitivity of p53-mutant containing UMSCC-1 cells, whereas a mild radioprotective phenotype on p53-wild type cell lines (UMSCC-6 and UMSCC-47) was seen [11]. Unlike Chk1 inhibition, there is currently limited evidence for the use of Wee1 inhibitors in radiosensitising HNSCC tumour models. However, one study performed in oral cavity HNSCC cell lines showed an increased radiosensitivity following Wee1 inhibition (MK-1775) associated with significantly increased apoptotic cell death [12]. Despite this, there are multiple completed or ongoing phase I clinical trials with Wee1 inhibition to investigate the maximum tolerated dose for future use in combination with the current standard chemoradiation regime (either cisplatin or cetuximab; NCT03028766, NCT02585973, NCT02555644) [13].

Interestingly, the potential for Chk1 and Wee1 inhibition to function in combination with PBT in radiosensitising HNSCC cells are currently lacking, and particularly to assess whether there is any impact of LET on the cellular response. One study has shown that the Chk1 inhibitor PF-00477736 effectively radiosensitises triple negative breast cancer (TNBC) cells to both X-rays and PBT, created through decreasing the proficiency of HR [14]. However, no studies examining Chk1 inhibition following PBT in HNSCC cell models are available, and also to our knowledge, there is no current evidence for the radiosensitising potential of Wee1 kinase inhibition in combination with PBT in any tumour type. In this study, we have characterised the radiosensitisation potential of both Chk1 and Wee1 checkpoint kinase inhibition (using MK-8776 and MK-1775, respectively) in response to X-rays and PBT (including an examination of both low and relatively high-LET protons) in 2D and 3D HNSCC models. We demonstrate that the radiosensitivity of HNSCC can be significantly enhanced following the inhibition of either Chk1 or Wee1, and that mechanistically this is associated with delayed DSB repair through reduced HR efficiency. Our findings suggest that this could represent a potential therapeutic avenue for optimising the treatment of HNSCC tumours using radiotherapy.

## Materials and methods

### Antibodies and inhibitors

Inhibitors for Chk1 (MK-8776) and Wee1 (MK-1775) were purchased from Selleck Chemicals (Munich, Germany). Antibodies against γH2AX (05-636; Merck-Millipore, Watford, UK), RAD51 (ab133534, Abcam, Cambridge, UK) were used for immunofluorescent staining, along with goat anti-mouse Alexa Fluor 555 and goat anti-rabbit Alexa Fluor 488 (Life Technologies, Paisley UK) secondary antibodies. The following primary antibodies were used for immunoblotting:- phosphorylated Chk1 (S296), Cdc2 (Y15), ATR (T1989), ATM (S1981) and Chk1 (S345), in addition to unmodified Chk1 and Wee1 (2349, 4539, 58014, 13050, 2348, 2360 and 4936, respectively; Cell Signaling Technology, Massachusetts, USA); ATM and ATR (ab78 and ab2905, respectively; Abcam, Cambridge, UK); PARP-1 (sc-53643; Santa-Cruz Biotechnology, Heidelberg, Germany) and β-actin (A5441; Merck-Sigma, Gillingham, UK). The following primary antibodies were used in the DNA fibre spreading assay:- rat anti-BrdU (347580, BD Bioscience) and mouse anti-BrdU (ab6326, Abcam, Cambridge, UK).

### Cell culture, siRNA transfections and irradiations

FaDu and A253 were purchased from ATCC (Teddington, UK) and cultured in Minimal Essential Medium (MEM) and McCoy’s 5A modified medium, respectively. UMSCC12 were kindly provided by Prof. T Carey (University of Michigan, USA) and cultured in Dulbecco’s Modified Eagle Medium (DMEM). All media was supplemented with 10% foetal bovine serum, 1x non-essential amino acids, 1x penicillin-streptomycin and 2 mM L-glutamine. For siRNA knockdowns, cells were seeded and after 24 h were transfected with a pool of four siRNA sequences targeting either CHEK1 or WEE1 (Horizon Discovery, Cambridge, UK), utilising RNAiMax (Life Technologies, Paisley, UK). Cells were treated with siRNA for 48 h before being utilised in immunoblotting and immunofluorescent staining, clonogenic survival assays, cell cycle analysis or spheroid growth assays.

X-ray irradiations were performed at a dose rate of ~3 Gy/min using a 150 kV CellRad+ X-ray irradiator (Faxitron Bioptics, Tucson, AZ, USA). PBT irradiations were performed using the MC-40 cyclotron utilising a 28 MeV beam at a dose rate of ~5 Gy/min. A Monte Carlo simulation has been developed of the MC-40 beamline used in these studies using Geant4 [15]. This model consists of an accurate geometry of the system and validated beam line transport. A large uniform beam was achieved by passing the 28 MeV protons through a 100 μm thick Tantalum foil located 3.2 m from the cells. Accounting for the vacuum window and ionisation chambers, the primary beam has a kinetic energy of 25.7 MeV incident upon the cell dish. The cell dish base is modelled as 1.2 mm of polystyrene and the LET is calculated from energy deposits in water directly after the cell dish base. For low-LET data, the mean kinetic energy incident upon the cell layer is 22.9 MeV and the LET calculated to be 2.7 keV/um. For the relatively high-LET data, 4.5 mm of Perspex was placed before the cell dish, and a beam of 3.8 MeV protons is incident upon the cells with a mean LET of 10.8 keV/um.

### Cell viability and clonogenic survival assays

For cell viability assays, 10,000 cells were treated with a serial dilution of either MK-8776 and MK-1775 (0.1–100 µM) in a 96-well plate 24 h after seeding, using either DMSO as a vehicle only control or hydrogen peroxide (10 mM) as a positive control. Cells were treated with these drugs for 72 h before viability was assessed. CellTiter Blue reagent (Promega, Wisconsin, USA) was added, cells were incubated at 5% CO_2_ at 37 °C for 2 h before measuring absorbance at a wavelength of 570 nm (A_570_), with the reference wavelength of 600 nm (R_600_). A_570_–R_600_ was calculated, and the survival of drug-treated cells relative to the DMSO control (after subtraction of the positive control) was determined.

For clonogenic survival assays, single cells were seeded in media containing either MK-8776 or MK-1775 for 16 h prior to irradiation, using DMSO as a vehicle only control. Plating efficiencies for the cells were as follows: FaDu and UMSCC12 ( ~30%) and A253 ( ~10%). The numbers of cells seeded were doubled for each increase in radiation dose to allow for plating efficiencies. Following irradiation, the media was replaced and colonies left to form for 7–14 days before staining with 20% methanol and 0.5% crystal violet for 1 h. Colonies were counted using the GelCount colony counter (Oxford Optronix, Oxford, UK), and the surviving fraction was then calculated as colonies per treatment versus colonies in respective unirradiated control.

### Spheroid growth assays

Cells were seeded at 500 cells/well in triplicate in ultra-low attachment plates (Greiner, Bio-One, Gloucestershire, UK) in 100 µl advanced DMEM F12 media containing 1% B-27 supplement, 0.5% N-2 supplement, 2 mM L-glutamine, 1x penicillin-streptomycin, 5 µg/ml heparin, 20 ng/µl epidermal growth factor (EGF) and 10 ng/µl fibroblast growth factor (FGF). Spheroids were left to form for 48 h before being treated with either MK-8776 or MK-1775, with DMSO as a vehicle only control, for 1 h. Immediately following irradiation, 60 µl of culture media was removed and replaced with 100 µl fresh media. Spheroids were then imaged at intervals up to day 13 post-seeding using the EVOS M5000 Imaging system (Life technologies, Paisley, UK). Spheroid diameter was determined using ImageJ, and converted to volume using the formula 4/3πr^3^.

### Patient-derived organoid generation and viability assays

Tissue samples were immediately transferred into a tube containing RPMI1640 medium complemented with 2.5% penicillin/streptomycin and 1% gentamicin, and stored at 4 °C until required. All the following processing was performed on ice. The tissue was cut into small pieces (∼1 mm^3^), and enzymatically digested in tissue dissociation mix, consisting of Advanced DMEM/F12 medium complemented with 100 µg/ml DNAse I, 100 µg/ml Dispase (Corning, New York, USA) and 10 µM Y-27632 dihydrochloride (Abmole, Texas, USA). After incubation at 37 °C for 30 min, enzymatic dissociation was stopped on ice by adding basal media, consisting of Advanced DMEM/F12, 1 M HEPES, 1% GlutaMax, 1 mM N-Acetyl-L-cysteine, 1% penicillin/streptomycin, 10 nM gastrin and 5 µM Y-27632 dihydrochloride. The suspension was strained over 500 µm and 40 µm filters (pluriSelect), with fragments within this range being employed for organoid cultures. Fragments were then plated and cultured in organoid medium, consisting of basal media further supplemented with 1% N-2 supplement, 2% B-27 supplement, 20 ng/ml FGF-2, 50 ng/ml EGF, 0.5 μM TGF-β RI kinase VI inhibitor (SB431542) and conditioned medium from the cell line CRL-2376™ (1:100, ATCC, Manassas, VA, USA), containing Wnt-3A, R-spondin and Noggin; with 250 µg/ml Amphotericin B added for the first three days of fragment culture only. At 80% confluency, cells were initially seeded at 2000 cells in a 15 μl dome of Matrigel before being left to grow. RNAseq was performed which revealed very low (<0.01%) contamination of organoids with immune, stromal and fibroblast cells.

For analysing radiosensitivity, 500 cells were seeded in a 5 µl dome of GelTrex (Life Technologies, Paisley, UK) in clear-bottom, black-walled 96-well plates. Organoids were left to form for 4 days before being treated for 16 h with either MK-8776 or MK-1775, with DMSO as a vehicle only control and hydrogen peroxide (10 mM) as a positive control. Following irradiation, organoids were left to grow for 4 days before viability was measured using CellTiter Glo (Promega, Wisconsin, USA) added 1:1 to the cell media. Plates were protected from light and shaken vigorously at 450 rpm for 5 min and left for a further 25 min before the luminescence was recorded at 1 second per well. Organoid viability was determined in the treated samples relative to the vehicle only control (and where the positive control was subtracted from both samples).

### Cell cycle analysis

Cells were harvested 24 h post-treatment and fixed in 70% ethanol for 1 h. Fixed samples were stained with propidium iodide (10 µg/ml) containing RNAse A (0.1 mg/ml) in PBT-T (0.05% Tween-20) for 1 h before being analysed via flow cytometry. A CytoFlex was used for data accumulation, using CytoExpert for further analysis (Beckman Coulter, Wycombe, UK).

### DNA fibre spreading assay

Replication stress was analysed using the DNA fibre spreading assay, specifically investigating replication speed. Cells were pulse labelled with IdU for 20 min then CIdU for 20 min. Following this, the cells were washed with ice cold PBS to stop replication and trypsinised. Cells were then lysed and spread onto microscope slides in spreading buffer (260 mM Tris-HCl (pH7.4), 50 mM EDTA, 17.3 mM SDS) for 2 min, before being tilted at a 45° angle. Once dried, cells were fixed in methanol/acetic acid (3:1) and dried overnight. Cells were denatured using HCl (2.5 M) for 1 h before being stained. Labelled fibres were visualised using an BX53FL Olympus Microscope with a 60x oil-immersion objective, and analysed using ImageJ software.

### Immunoblotting and immunofluorescent staining

Whole cell extracts were prepared from HNSCC cells and analysed by immunoblotting as previously described [16]. For immunofluorescent staining of γH2AX and RAD51 foci, cells were grown on 13 mm coverslips. Following irradiation (4 Gy), cells were left to repair for the required time in 5% CO_2_ at 37 °C prior to fixing with 10% formalin and staining as previously described [16].

### Enzyme-modified neutral comet assay

The levels of DSB and CDD (specifically alkali-labile sites and oxidative DNA base damage recognised by APE1 and the DNA glycosylases OGG1 and NTH1, respectively) were analysed using the enzyme-modified comet assay, as previously described [17]. In brief, and following irradiation (4 Gy), cells were embedded in 1% low melting-point agarose on precoated microscope slides and left to repair in a humidified chamber at 37 °C for the required time before being lysed for at least 1 h in cold lysis buffer containing 2.5 M NaCl, 100 mM EDTA disodium salt, 10 mM Tris base, 1% N-lauroylsarcosine, 1% DMSO and 1% (v/v) Triton X-100 (pH 9.5). For the analysis of DSBs, slides were incubated with enzyme reaction buffer alone (40 mM HEPES-KOH, 100 mM KCl, 0.5 mM EDTA and 0.2 mg/ml BSA, pH 8.0) whereas for CDD slides were incubated in buffer containing 5 pmol OGG1, 6 pmol NTH1 and 0.6 pmol APE1 for 45 min at 37 °C. These enzymes concentrations were initially determined through titration experiments comparing low and high-LET protons, ensuring that only CDD was revealed following high-LET proton conditions [17]. Slides were then placed in cold electrophoresis buffer (1x TBE, pH 8.3) leaving the DNA to unwind for 30 min, prior to electrophoresis at 25 V, ~15 mA for 25 min. Slides were washed three times with cold PBS before being dried overnight. Following rehydration in water (pH 8) for 30 min, DNA was stained with SYBR Gold (Life Technologies, Paisley, UK) 1:20,000 (in water, pH 8) for 30 min and dried overnight. Comets were visualised using an BX53FL Olympus Microscope with a 10x objective, and 100 cells per condition analysed using the Komet 6.0 image analysis software (Andor Technology, Belfast, Northern Ireland) to determine % tail DNA values.

### Statistical analysis

All experiments were performed in at least triplicate as separate, independent, biological experiments. Statistical analysis of clonogenic survival was performed using CFAssay package for R using the linear quadratic (LQ) model [18]. Dose enhancement ratios were calculated from linear quadratic fitting at a surviving fraction of 0.5, comparing the doses required to achieve the same survival in the DMSO only versus to the inhibitor treated cells. Statistical analysis of DNA damage and repair (γH2AX/RAD51 foci and enzyme-modified comet assays) and cell cycle data was performed using a one-sample *t* test, with DNA fibre data being analysed with a Mann-Whitney test.

## Results

### Chk1 and Wee1 inhibition radiosensitises HNSCC cells to X-ray irradiation

Initially, we investigated the cytotoxic effects of the Chk1 inhibitor (MK-8776) and Wee1 inhibitor (MK-1775) in FaDu, UMSCC12 and A253 HNSCC cell lines (Supplementary Fig. 1A–C). MK-8776 seemed to be well tolerated in FaDu and UMSCC12 cells up to 10 μM, whereas A253 cells showed evidence of toxicity at 0.1 μM and above. MK-1775 seemed to be well tolerated by all three cell lines up to a dose of 1 μM. These viability assays represent the tolerability of the cells when treated as a monolayers. Under clonogenic assay conditions, where cells are seeded and treated individually, this revealed that both UMSCC12 and A253 tolerated 1 μM MK-8776, whilst FaDu were able to tolerate up to 10 μM (Supplementary Fig. 1D–F). Additionally, all three cell lines maintained significant cellular survival following 0.2 μM MK-1775 (Supplementary Fig. 1G–I). These concentrations were therefore taken forward for clonogenic survival analysis. We also validated the effectiveness of the inhibitors in supressing kinase activity in HNSCC cell lines following X-ray irradiation, by analysing the phosphorylation of either the auto-phosphorylation site on Chk1 (pS296) or the downstream target of CDC2 (pY15). This demonstrated a dose-dependent decrease in kinase activity which was maximal using ~2 µM of both MK-8776 and MK-1775 in monolayer treated cells (Supplementary Figs. 2A–D, 3A–D and 4A–D).

**Fig. 1:**
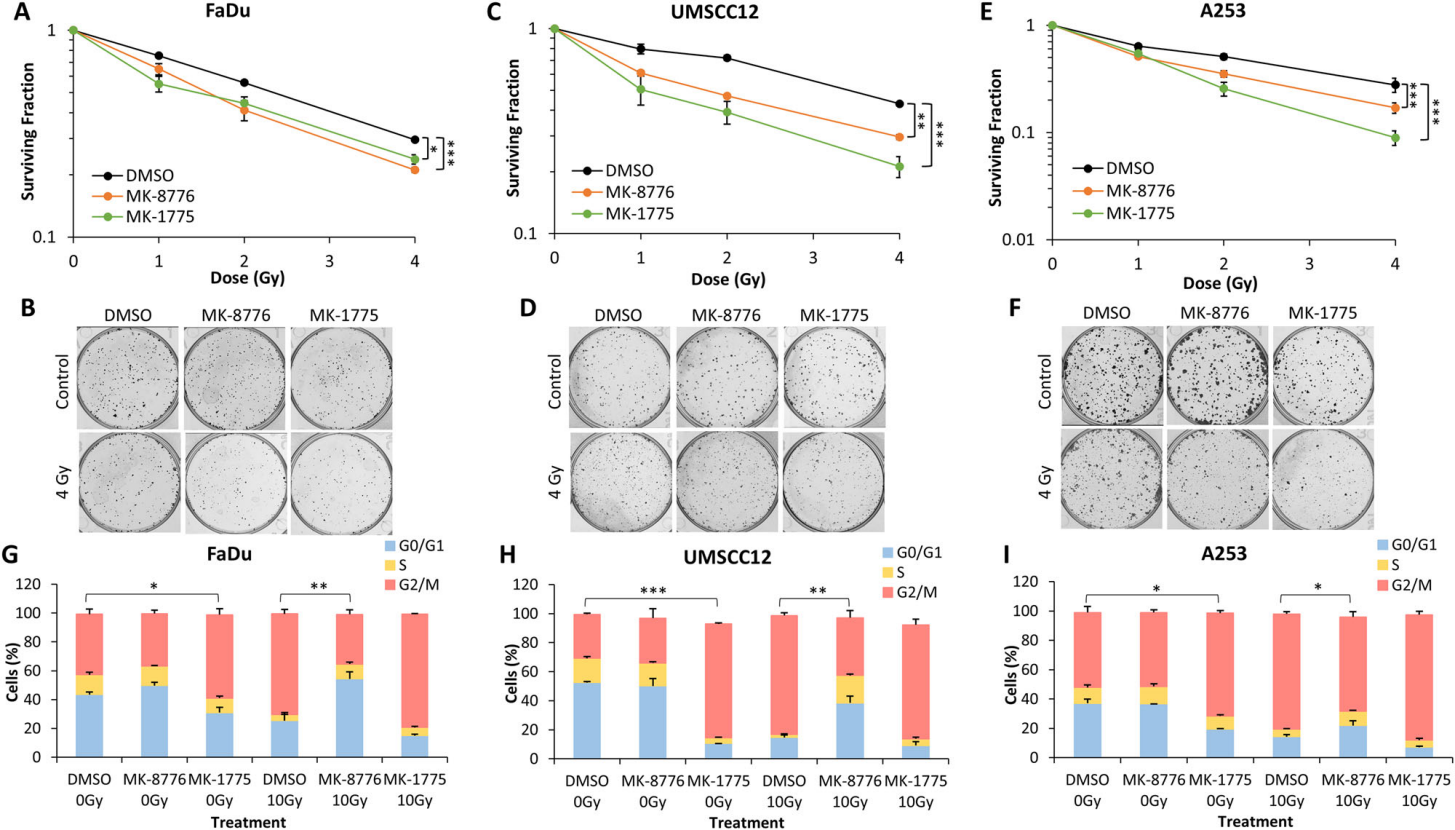
Inhibition of Chk1 or Wee1 results in increased radiosensitivity of HNSCC cells to X-ray radiation and abrogation of radiation-induced G_2_/M phase arrest. (**A**, **B**) FaDu, (**C**, **D**) UMSCC12 or (**E**, **F**) A253 cells were treated with either 1 μM MK-8776 (10 μM for FaDu) or 0.2 μM MK-1775 for ~16 h prior to exposure to X-ray radiation, and clonogenic survival of the cells was analysed from three biologically independent experiments. Shown is the mean surviving fraction±SE. *p < 0.01, **p < 0.002, ***p < 0.0001 as analysed by the CFAssay. Representative images of (**B**) FaDu, (**D**) UMSCC12 and (**F**) A253 show colony formation in unirradiated (control) cells and following 4 Gy X-ray radiation (four times number of cells seeded). (**G**) FaDu, (**H**) UMSCC12 or (**I**) A253 were treated with either 10 μM MK-8776 or 2 μM MK-1775 ~16 h prior to exposure to 10 Gy X-ray radiation and samples harvested 24 h later for cell cycle analysis by flow cytometry. *p < 0.05, **p < 0.005, ***p < 0.0001, as analysed by a one sample *t* test.

We subsequently investigated the effects of MK-8776 and MK-1775 in combination with X-ray irradiation on the clonogenic survival of HNSCC cells. Pre-treatment of cells with either 1 μM MK-8776 (10 μM used in FaDu only) or 0.2 μM MK-1775 led to significantly decreased survival of FaDu, UMSCC12 and A253 cells in response to X-ray irradiation (Fig. 1A–F). Dose enhancement ratios calculated at 50% survival (DER_50_) ranged from 1.26–1.64 (MK-8776) and 1.38–1.81 (MK-1775; Table 1). The linear-quadratic (LQ) curves are also shown (Supplementary Fig. 5A–C). All three cell lines were shown to express Chk1 and Wee1 proteins, and both these and associated key DNA damage response proteins were activated following X-ray irradiation (Supplementary Fig. 6, and 7A–G). To support the inhibitor-radiation observations, an siRNA knockdown of either CHEK1 or WEE1 was performed. Similar to the targeted inhibitors, an absence of either Chk1 or Wee1 was shown to radiosensitise FaDu and UMSCC12 cells using clonogenic survival assays (Supplementary Figs. 8A–F and 9A–D), and where DER_50_ values of 1.44–1.53 and 1.37–1.46, respectively were observed compared to the non-targeting siRNA control (Supplementary Table 1). As both Chk1 and Wee1 kinases have major characterised roles in mediating G_2_/M checkpoint arrest, we investigated the cell cycle distribution of cells following treatment with the inhibitors alone and in combination with X-ray radiation. All three HNSCC cell lines (FaDu, UMSCC12 and A253) demonstrated an increase in G_2_/M arrest in response to X-ray irradiation, and which was overcome using MK-8776 compared to the DMSO irradiated control (Fig. 1G–I). Interestingly, MK-1775 alone caused a significant increase in the G_2_/M population of cells suggesting an alteration of cell cycle regulation. However, MK-1775 appeared to have no impact on radiation-induced arrest, at least at the 24 h time point investigated. These findings were supported by cell cycle analysis following siRNA knockdown of either CHEK1 or WEE1, in both FaDu and UMSCC12 (Supplementary Fig. 8G, H).

**Table 1:** Dose enhancement ratios (DER) at 50% survival for HNSCC cell lines following Chk1 or Wee1 inhibition with X-ray radiation.

DER_50%_	Cell Line		
Treatment	FaDu	UMSCC12	A253
MK-8776	1.26	1.35	1.64
MK-1775	1.38	1.81	1.61

### Chk1 and Wee1 inhibition reduces DSB repair efficiency following X-ray irradiation

**Fig. 2:**
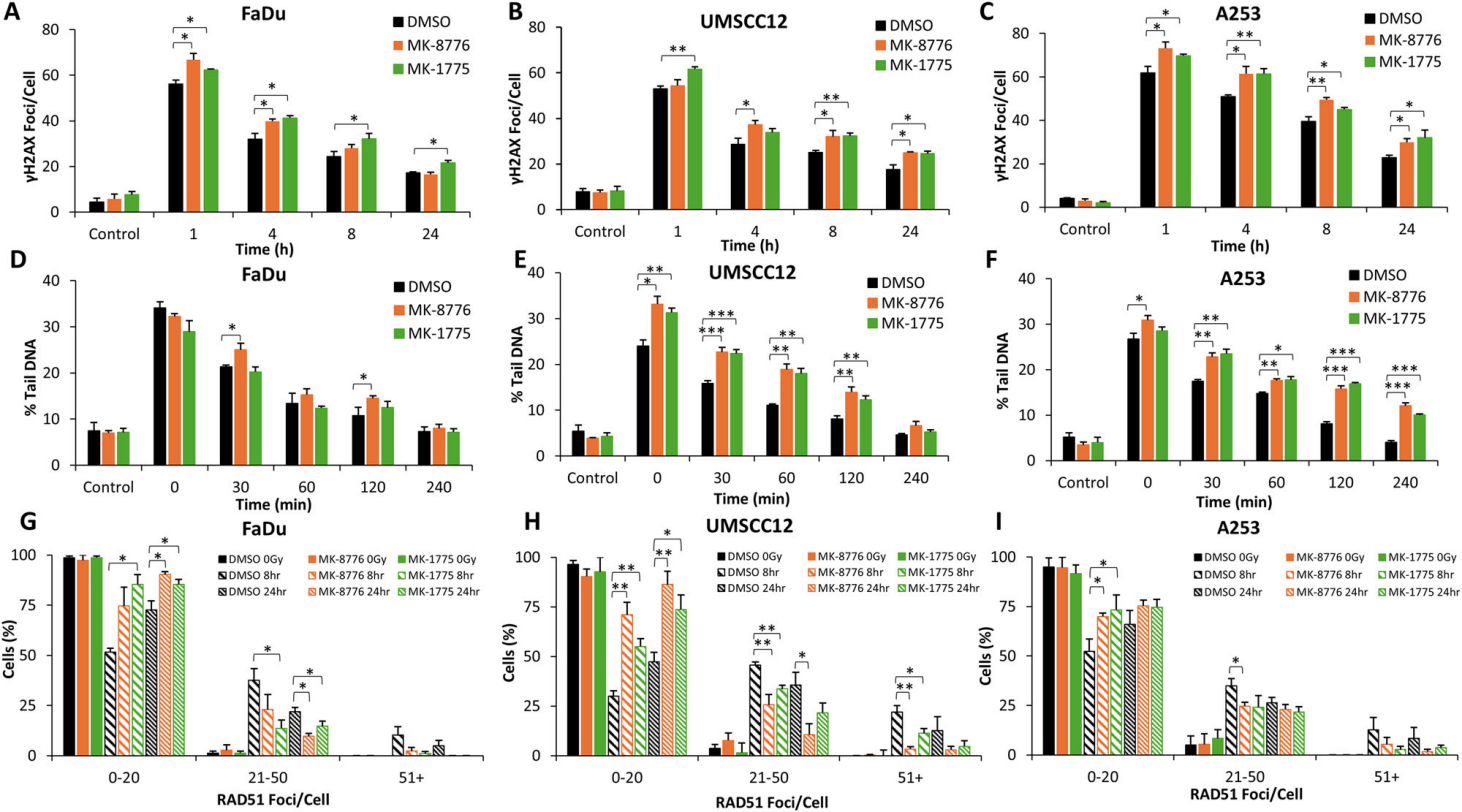
Chk1 or Wee1 inhibition causes increased persistence of X-ray radiation-induced DSBs. **A**, **D**, **G** FaDu, (**B**, **E**, **H**) UMSCC12 or (**C**, **F**, **I**) A253 cells were treated with either 1 μM MK-8776 (10 μM for FaDu) or 0.2 μM MK-1775 for ~16 h prior to exposure to 4 Gy X-Ray radiation. DNA DSB damage was measured at various timepoints post-irradiation using (**A**–**C**) γH2AX foci as a marker using immunofluorescence microscopy or (**D**–**F**) neutral comet assays. (**G**–**I)** RAD51 foci as an indicator of HR repair were also measured using immunofluorescence microscopy, with the percentage of cells containing a certain number of foci being categorised. Data are shown as mean ± SE. *p < 0.05, **p < 0.01, ***p < 0.001, as analysed by a one sample *t* test.

Using γH2AX foci as a marker of DSB damage, we show that FaDu, UMSCC12 and A253 cells demonstrate a statistically significant increase in DSBs at 1 h post-irradiation in the presence of MK-8776 or MK-1775 versus the DMSO control (Fig. 2A–C and Supplementary Fig. 10A–C). The levels of DSBs then persist up to 24 h post-irradiation (apart from MK-8776 in FaDu cells which significantly persists for up to 4 h post-irradiation), demonstrating deficiencies in DSB repair with the inhibitor-radiation combinations. To support these observations, γH2AX foci analysis was performed following siRNA knockdown of either CHEK1 or WEE1, which similar to the inhibitors demonstrated an increased persistence of DNA damage for up to 24 h post-irradiation, in both FaDu and UMSCC12 cell lines (Supplementary Fig. 11A–D). To validate DSB persistence, we utilised the neutral comet assay to investigate the repair of DSBs directly. In both UMSCC12 and A253 cell lines, there was significant increase in the levels and persistence of DSBs following treatment with either MK-8776 or MK-1775 up to 120 min post-X-ray irradiation (which increased to 240 min post-irradiation in A253 cells; Fig. 2E, F and Supplementary Fig. 12B, C). Interestingly, there was no dramatic increase or persistence of DSBs in FaDu cells with either MK-8776 or MK-1775 post-irradiation, although there was a tendency for higher DSB levels in Chk1-inhibited cells which was significant at 30 and 120 min post-irradiation (Fig. 2D and Supplementary Fig. 12A). Cumulatively, this suggests that Chk1 and Wee1 inhibition cause delays in the repair of radiation-induced DSBs. We next investigated the proficiency of HR using RAD51 foci as a marker. We observed a higher percentage of cells with increased numbers of RAD51 foci in the DMSO control treated cells analysed 8 and 24 h post-irradiation, compared to cells irradiated in the presence of the inhibitors (Fig. 2G–I and Supplementary Fig. 13A–C). This demonstrates that Chk1 and Wee1 inhibition reduces the efficiency of HR, contributing to the increased persistence of DSBs. This evidence was further supported by observations that the majority of cells (FaDu and UMSCC12) treated with siRNA knockdown of either CHEK1 or WEE1 maintained low levels of RAD51 foci at both 8 and 24 h post-irradiation, compared to siRNA non-targeted treated cells (Supplementary Fig. 14A–D). We also analysed PARP-1 cleavage as a marker of apoptosis but which didn’t reveal any evidence for significant increases in apoptotic cell death with the inhibitor-radiation combination (Supplementary Fig. 15A and 16A, B). Additionally through DNA fibre analysis, whilst the inhibitors alone suppressed DNA replication speeds, the combination of MK-8776 or MK-1775 with X-ray irradiation was additive but not synergistic in supressing DNA replication (Supplementary Fig. 15B).

### Chk1 and Wee1 inhibition radiosensitises HNSCC cells to both low- and relatively high-LET PBT

**Fig. 3:**
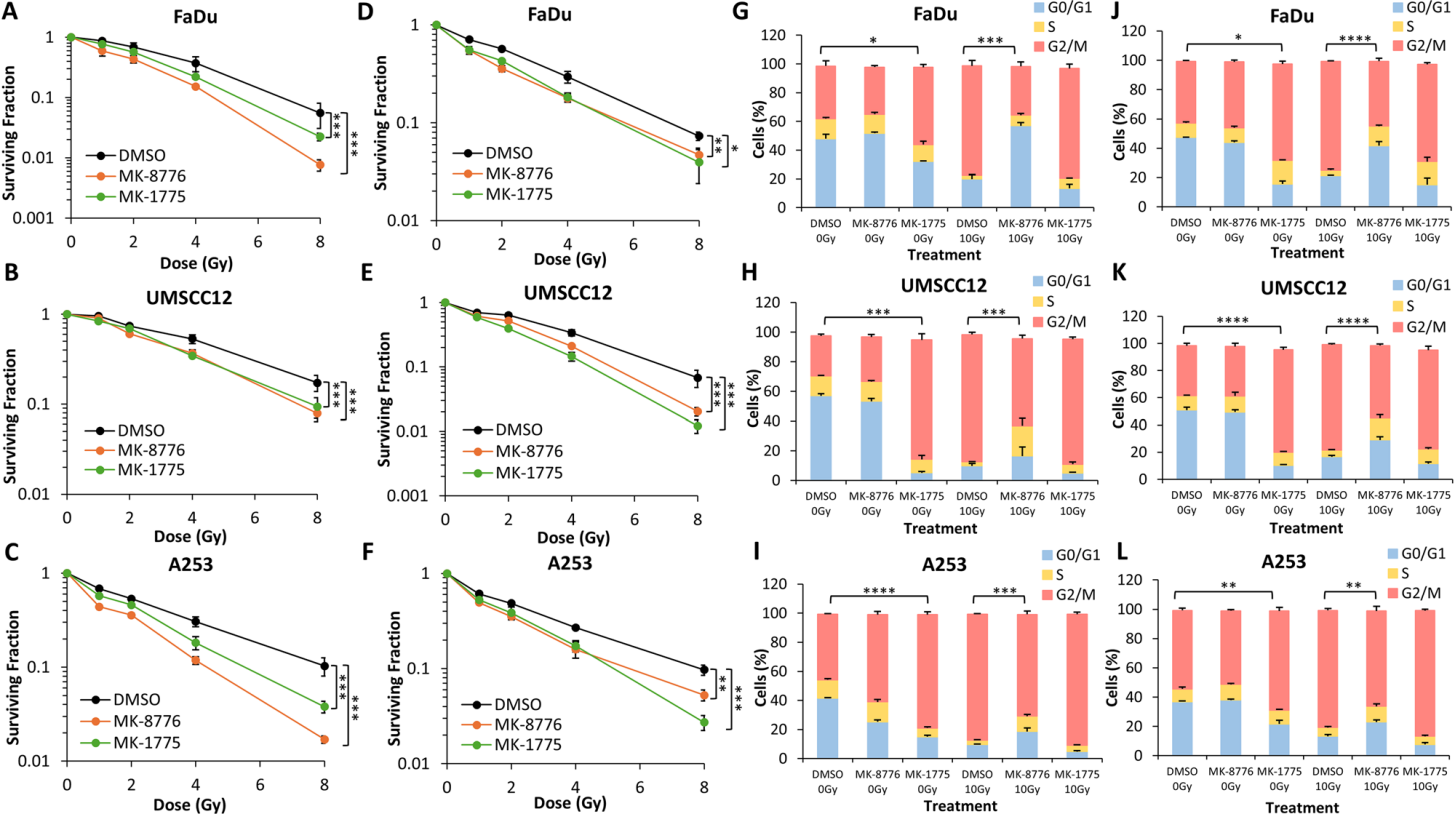
Inhibition of Chk1 or Wee1 results in increased radiosensitivity of HNSCC cells to both low and relatively high-LET PBT and abrogation of radiation-induced G_2_/M phase arrest. **A**, **D** FaDu, (**B**, **E**) UMSCC12 or (**C**, **F**) A253 cells were treated with either 1 μM MK-8776 (10 μM for FaDu) or 0.2 μM MK-1775 for ~16 h prior to exposure to either (**A**–**C**) low-LET or (**D**–**F**) relatively high-LET PBT and clonogenic survival of the cells was analysed from three biologically independent experiments. Shown is the mean surviving fraction ± SE. *p < 0.02, **p < 0.002, **p < 0.0001 as analysed by the CFAssay. (**G**, **J**) FaDu, (**H**, **K**) UMSCC12 or (**I**, **L**) A253 were treated with either 10 μM MK-8776 or 2 μM MK-1775 ~16 h prior to exposure to 10 Gy (**G**–**I**) low-LET or (**J**–**L**) relatively high-LET PBT and samples harvested 24 h later for cell cycle analysis by flow cytometry. *p < 0.05, **p < 0.01, ***p < 0.002, ****p < 0.0001, as analysed by a one sample *t* test.

Next, we investigated the effect of inhibiting either Chk1 or Wee1 kinases in HNSCC cells in combination with PBT, with cells positioned either in the entrance plateau at low-LET (2.7 keV/µm), or within the Bragg peak at relatively high-LET (10.8 keV/µm). Pre-treatment with either MK-8776 or MK-1775 significantly reduced the survival of FaDu, UMSCC12 and A253 cells in response to low-LET PBT (Fig. 3A–C and Supplementary Fig. 17A–C), with DER_50_ values of 1.44–1.81 and 1.29–1.43 for MK-8776 and MK-1775, respectively (Table 2). MK-8776 or MK-1775 were also shown to significantly enhance the radiosensitivity of FaDu, UMSCC12 and A253 cells following high-LET PBT (Fig. 3D–F and Supplementary Fig. 17D–F), and where DER_50_ values of 1.37–1.56 and 1.40–1.78 for MK-8776 and MK-1775, respectively were observed (Table 2). As demonstrated previously, Wee1 inhibition alone appeared to cause an accumulation of HNSCC cells in G_2_/M suggesting altered cell cycle regulation (Fig. 3G–L). Both low-LET and high-LET PBT induced significant G_2_/M arrest, whilst this arrest was observed to be overcome in the presence of MK-8776 in all three cell lines (Fig. 3G–L). Nevertheless, the impact of both inhibitors in abrogating the cell cycle post-irradiation was independent of PBT LET.

**Table 2:** Dose enhancement ratios (DER) at 50% survival for HNSCC cell lines following Chk1 or Wee1 inhibition with PBT.

DER_50%_	Cell Line		
Treatment	FaDu	UMSCC12	A253
Low-LET MK-8776	1.59	1.44	1.81
Low-LET MK-1775	1.29	1.43	1.39
High-LET MK-8776	1.56	1.37	1.52
High-LET MK-1775	1.42	1.78	1.40

### Chk1 and Wee1 inhibition reduces DSB repair following both low- and relatively high-LET PBT

**Fig. 4:**
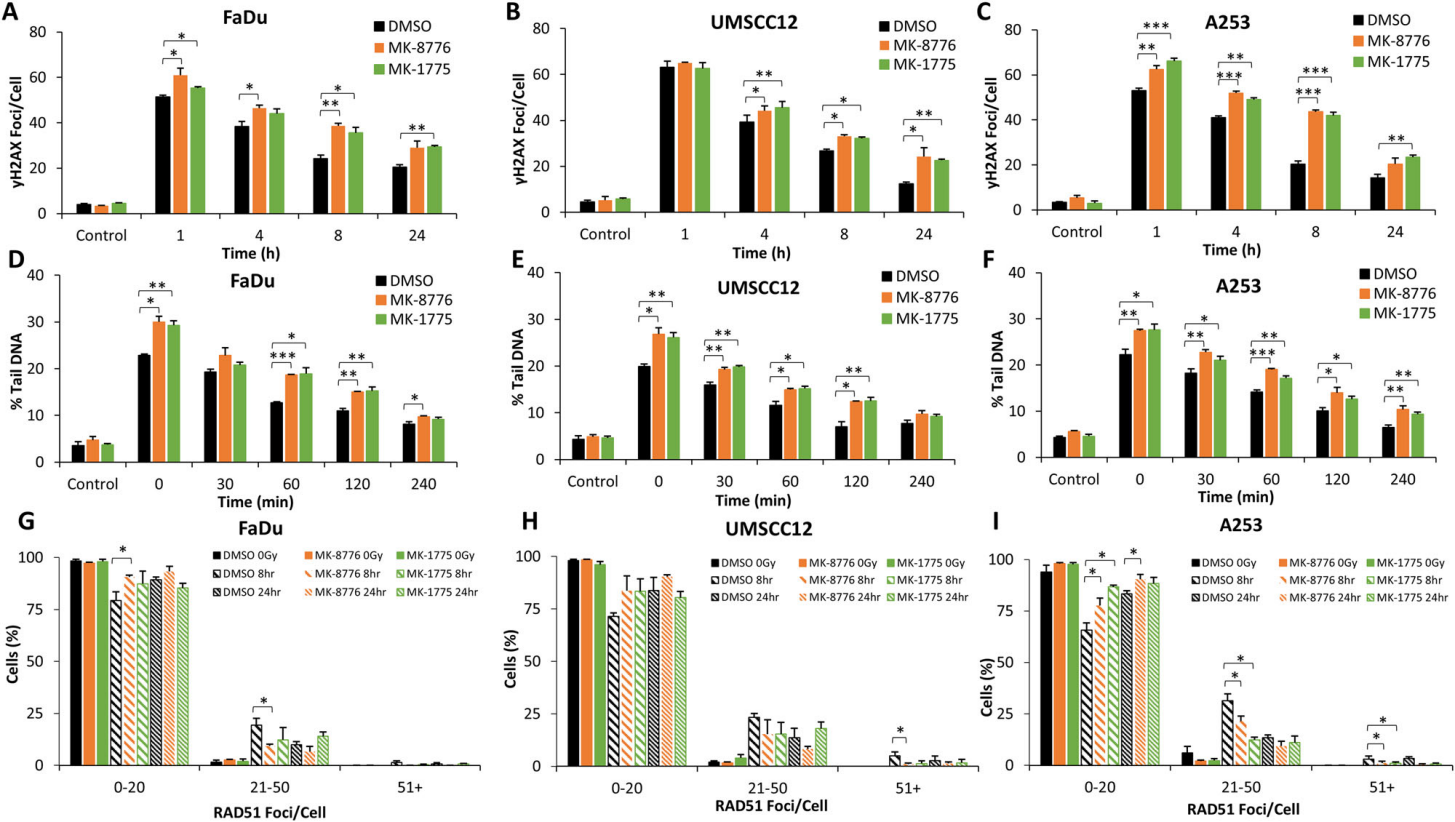
Chk1 or Wee1 inhibition causes increased persistence of low-LET PBT-induced DSBs. **A**, **D**, **G** FaDu, (**B**, **E**, **H**) UMSCC12 or (**C**, **F**, **I**) A253 cells were treated with either 1 μM MK-8776 (10 μM for FaDu) or 0.2 μM MK-1775 for ~16 h prior to exposure to 4 Gy low-LET PBT. DNA DSB damage was measured at various timepoints post-irradiation using (**A**–**C**) γH2AX foci as a marker using immunofluorescence microscopy or (**D**–**F**) neutral comet assays. RAD51 foci as an indicator of HR repair were also measured using immunofluorescence microscopy, with the percentage of cells containing a certain number of foci being categorised (**G**–**I**). Data are shown as mean ± SE. *p < 0.05, **p < 0.01, ***p < 0.001, as analysed by a one sample *t* test.

Through analysis of γH2AX foci as a marker of DSB damage, we demonstrate that after 1 h post-irradiation with low-LET PBT in the presence of MK-8776 and MK-1775 there is a significant increase in the levels of DSBs in FaDu and A253 cells, versus DMSO-control irradiated cells. This increased level of DSBs in the presence of the checkpoint inhibitors persisted for up to 24 h post-irradiation in all the three cell lines (Fig. 4A–C and Supplementary Fig. 18A–C). Similarly, and using neutral comet assays to directly detect DSBs, this also showed that the repair of low-LET PBT-induced DSBs was reduced up to 120–240 min post-irradiation in the presence of MK-8776 and MK-1775 (Fig. 4D–F and Supplementary Fig. 19A–C). On exploring RAD51 foci as a marker of HR repair, interestingly there was an apparent overall reduced numbers of HNSCC cells with high numbers of RAD51 foci following PBT as compared to X-rays. Nevertheless, we observed that there was a reduced percentage of FaDu and UMSCC12 cells with high numbers of RAD51 foci at 8 and 24 h post-irradiation in the MK-8776 and MK-1775 treated conditions, compared to the DMSO-irradiated controls, but which was only statistically different in the presence of MK-8776 (Fig. 4G, H and Supplementary Fig. 20A, B). In contrast, both MK-8776 and MK-1775 caused significant decreases in A253 cells with high numbers of RAD51 foci at 8 and 24 h post-irradiation, compared to the DMSO-irradiated controls (Fig. 4I and Supplementary Fig. 20C). This reduction in RAD51 foci accumulation indicates reduced efficiency in the repair of DSBs through HR in HNSCC cells in the presence of Chk1 and Wee1 inhibition following low-LET PBT.

**Fig. 5:**
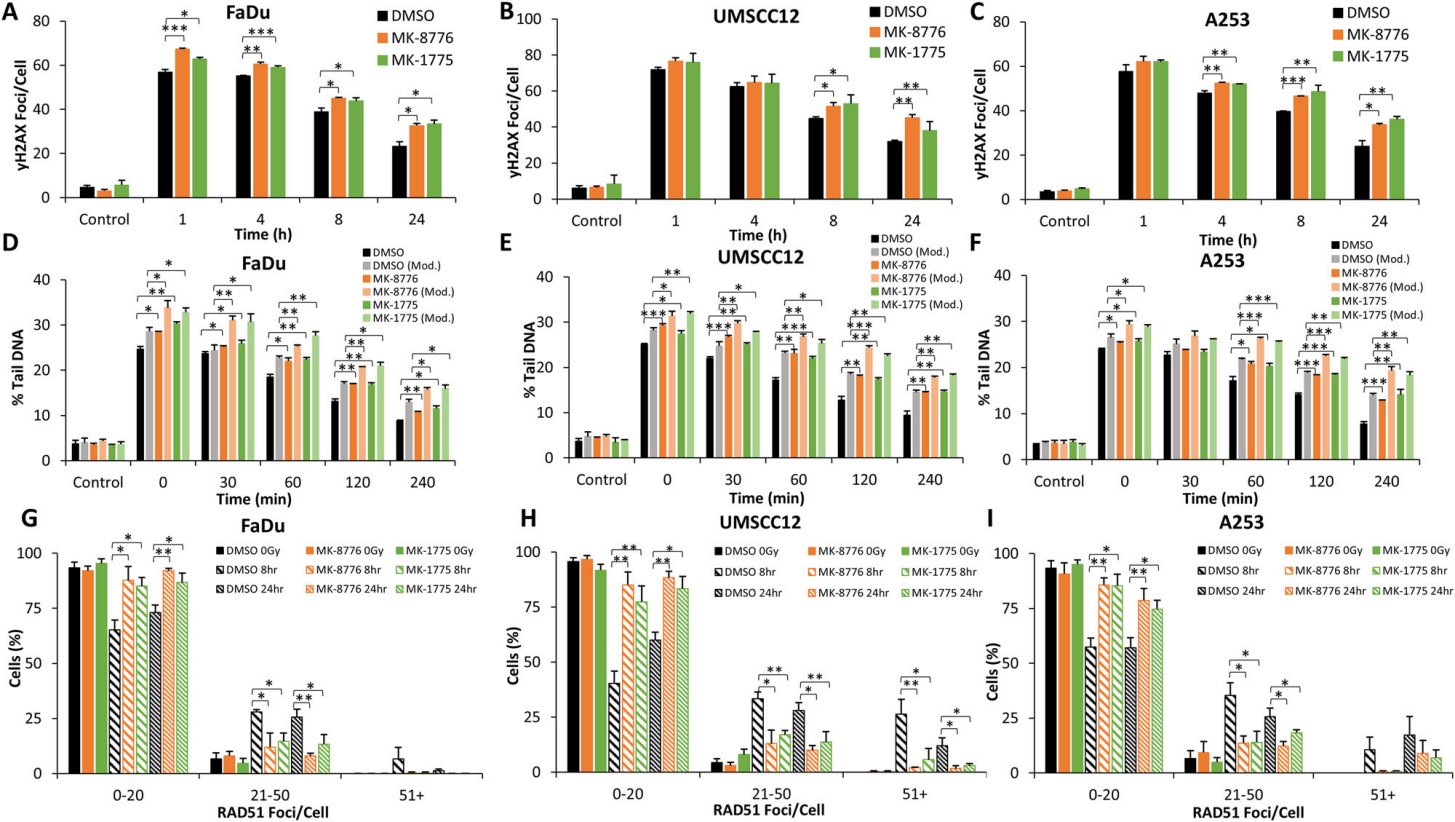
Chk1 or Wee1 inhibition causes increased persistence of high-LET PBT-induced DSBs. **A**, **D**, **G**) FaDu, (**B**, **E**, **H**) UMSCC12 or (**C**, **F**, **I**) A253 cells were treated with either 1 μM MK-8776 (10 μM for FaDu) or 0.2 μM MK-1775 for ~16 h prior to exposure to 4 Gy relatively high-LET PBT. DNA DSB damage was measured at various timepoints post-irradiation using (**A**–**C**) γH2AX foci as a marker using immunofluorescence microscopy. DNA damage was also analysed using enzyme modified neutral comet assays (**D**–**F**), through the incubation of cellular DNA in the absence (DSBs only) or presence (DSBs and CDD) of recombinant enzymes APE1, NTH1 and OGG1. RAD51 foci as an indicator of HR repair were also measured using immunofluorescence microscopy, with the percentage of cells containing a certain number of foci being categorised (**G**–**I**). Data are shown as mean ± SE. *p < 0.05, **p < 0.01, ***p < 0.001, as analysed by a one sample *t* test.

On mechanistic analysis of the cellular response to relatively high-LET PBT, we observed that γH2AX as a DSB marker was increased at 1 h post-irradiation in FaDu, and significantly persisted for at least 24 h post-irradiation, in all three cell lines in combination with either MK-8776 or MK-1775 compared to the DMSO controls (Fig. 5A–C and Supplementary Fig. 21A–C). To directly correlate this data with DSB levels, we utilised the neutral comet assay, but also employed an enzyme modified version of the assay to specifically and additionally detect the levels and repair of CDD (consisting of multiple DNA lesions in close proximity, including DNA strand breaks plus alkali-labile sites and oxidative DNA base damage) induced by high-LET PBT. In the absence of enzyme modification, and similar to the γH2AX foci data, this revealed a statistically significant delayed repair of DSBs following pre-treatment with either MK-8776 or MK-1775 compared to the DMSO controls (Fig. 5D–F; compare black, dark orange and dark green bars and Supplementary Fig. 22A–C). Following enzyme modification, this also showed significantly increased persistence of DNA damage following MK-8776 or MK-1775 pre-treatment compared to the DMSO controls (Fig. 5D–F; compare grey, light orange and light green bars and Supplementary Fig. 22D–F), although this difference did not appear to exceed that observed in the absence of enzyme modification. This data therefore indicates that the inhibitors mainly influence and supress the repair of DSB, rather than CDD. Through investigating RAD51 foci as a marker of the HR response, and similar to data acquired using X-ray irradiation, it was evident that there are an increase in the percentage of cells with a higher number of RAD51 foci in the DMSO irradiated conditions, compared to the cells pre-treated with either MK-8776 or MK-1775, at both 8 and 24 h post-irradiation (Fig. 5G–I and Supplementary Fig. 23A–C). This suggests reduced HR efficiency of the repair of DSBs with Chk1 and Wee1 inhibition following high-LET PBT.

### Chk1 and Wee1 inhibition radiosensitises patient-derived HNSCC organoids to X-rays and PBT

**Fig. 6:**
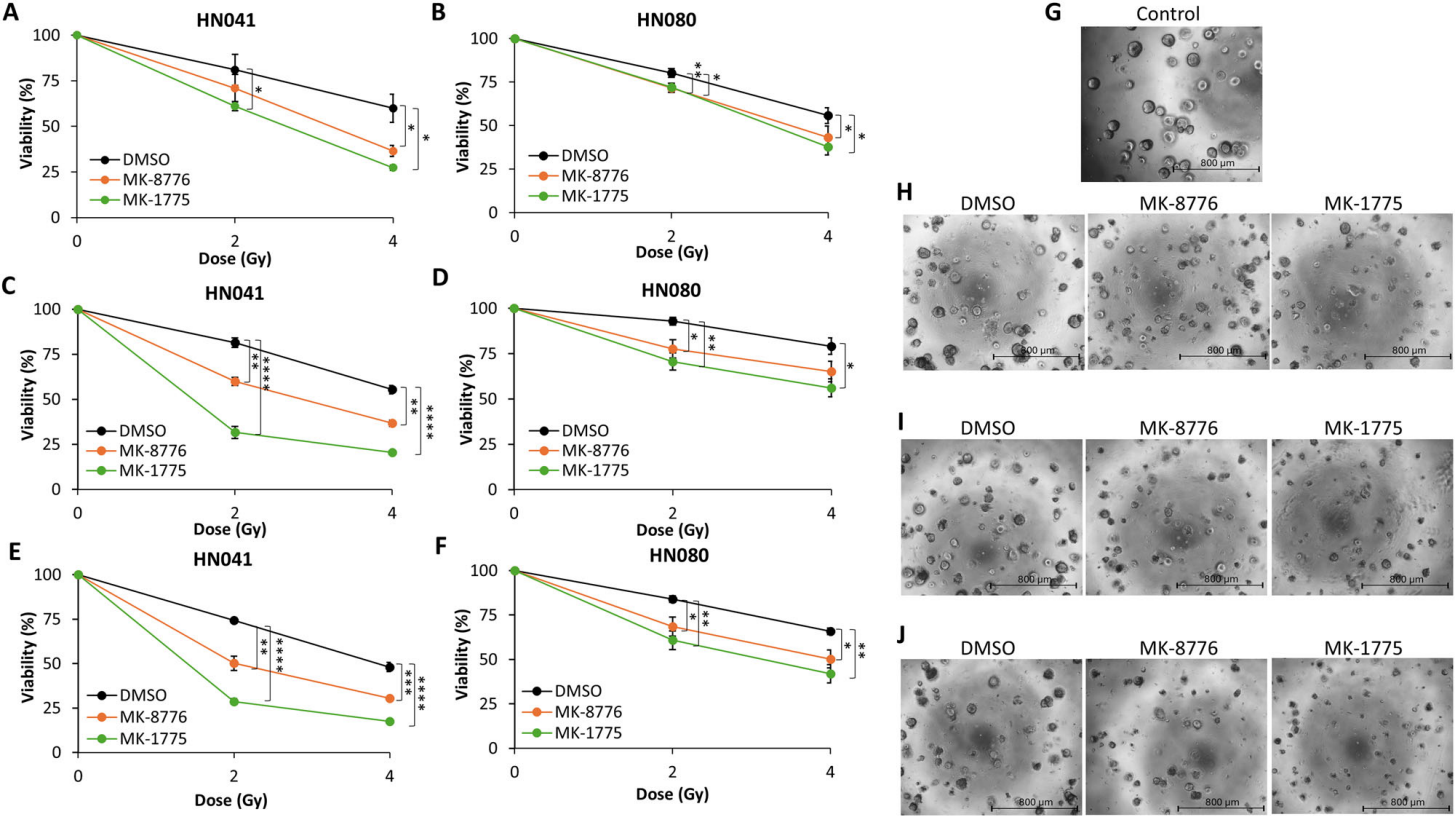
Chk1 or Wee1 inhibition increases radiosensitivity of patient-derived HNSCC organoids to X-rays and PBT. HN041 or HN080 patient-derived organoid models were pre-treated with either 2 μM MK-8776 or 0.4 μM MK-1775 for ~16 h prior to exposure to (**A**, **B**, **H**) X-Rays, (**C**, **D**, **I**) low-LET PBT or (**E**, **F**, **J**) relatively high-LET PBT. Cell survival was analysed using cell viability assays 4 days post-irradiation. Representative images of HN041 organoid model show the unirradiated (control) cells (**G**) and following 4 Gy (**H**) X-Rays, (**I**) low-LET PBT or (**J**) relatively high-LET PBT. Scale bar 800 µm. Data shown are mean cell survival ± SE. *p < 0.05, **p < 0.01, ***p < 0.002, ****p < 0.0002 as analysed by a one sample *t* test.

Following confirmation that MK-8776 and MK-1775 effectively radiosensitise HNSCC cell lines to X-rays and PBT (both at low- and high-LET), we proceeded to explore the effects of the drug-radiation combination on more translationally relevant 3D models. Firstly, we established that there was a significant reduction in growth and therefore enhanced radiosensitisation of FaDu cells grown as 3D spheroid models to X-rays following downregulation of CHEK1 and WEE1 using siRNA (Supplementary Fig. 24A–C). Additionally, FaDu and A253 HNSCC spheroids were significantly radiosensitised to both X-rays and low-LET PBT in the presence of the inhibitors MK-8776 and MK-1775 (Supplementary Fig. 25A–F and 26A–F). More importantly, utilising two patient-derived HNSCC organoid models treated with MK-8776 or MK-1775 prior to irradiation, we clearly demonstrate that organoid viability is significantly reduced with increasing doses of either X-rays (Fig. 6A, B, G, H), low-LET PBT (Fig. 6C, D, I) or high-LET PBT (Fig. 6E, F, J) compared to the DMSO control treated cells. This clearly demonstrates the radiosensitising potential of MK-8776 and MK-1775 in patient-derived HNSCC models in response to both X-rays and PBT of increasing LET.

## Discussion

Ionising radiation, including X-rays and PBT, is known to induce cell cycle arrest at the G_2_/M checkpoint to aid cellular repair of the DNA damage induced and to promote cell survival. Therefore, essential protein kinases involved in orchestrating this arrest, namely Chk1 and Wee1, have been suggested as targets to enhance the sensitivity of tumour cells to radiotherapy (reviewed in [19]). The inhibitors used in our study (MK-8776 and MK-1775) have been found to be effective radiosensitisers in various tumour types, including glioblastoma, lung, bladder and TNBC [10, 20–24]. Moreover, it has been previously shown that alternative Chk1 inhibitors (PF-00477736, LY2606368, CCT244747 and MK-8776) plus some limited evidence using the Wee1 inhibitor MK-1775 can effectively radiosensitise HNSCC cells to X-ray radiation [9, 11, 12, 25–29]. However in this study, we now clearly expand on this evidence base by demonstrating that Chk1 and Wee1 inhibition through MK-8776 and MK-1775, respectively are not only effective at enhancing the sensitivity of cell lines, spheroids and patient-derived organoid models of HNSCC to X-ray irradiation, but also can increase the efficacy of PBT at both low and relatively high-LET relative to the Bragg peak.

It has been previously observed that both HPV-positive and HPV-negative HNSCC cells (UMSCC-47, UDSCC-2, UTSCC-45, 93-VU-147T, FaDu and Cal33) display increased sensitivity to X-ray irradiation following pre-treatment with the Chk1 inhibitor PF-00477736, in conjunction with an abrogation of the radiation-induced G_2_/M arrest [9]. Another study performed with the Wee1 inhibitor MK-1775 in UMSCC-47, SSC-25, SSC-15 and Cal-27 cell lines showed that this significantly reduced the viability of the cells to X-rays [12]. Interestingly, a limited study observed that both MK-8776 and MK-1775 increased sensitivity of FaDu cells to irradiation, although it was suggested that Wee1 inhibition through MK-1775 was marginally the more effective approach for radiosensitisation (DER values of 1.26 and 1.49 for MK-8776 and MK-1775, respectively) [10]. This is in support of our study, as we demonstrated similar enhancements of radiosensitivity of FaDu cells to X-rays in the presence of both MK-8776 and MK-1775 (DER values of 1.26 and 1.38, respectively). Enhanced radiosensitivity of UMSCC12 cells to Wee1 versus Chk1 inhibition was also evident from our data, although A253 cells appeared to show similar radiosensitisation to both the inhibitors in combination with X-rays.

Our study shows an increase in the induction and persistence of X-ray-induced DSBs (as revealed by γH2AX foci and neutral comet assays) in FaDu, UMSCC12 and A253 cells in the presence of MK-8776 and MK-1775. Similar effects have also been seen in UMSCC-1 cells following Chk1 inhibition (AZD7762) at 24 and 48 h post-irradiation through increased tail moments in neutral comet assays compared to irradiation alone [11]. We further show that pre-treatment with both MK-8776 and MK-1775 results in a marked decrease in the percentage of cells with high numbers of RAD51 foci at both 8 and 24 h post-irradiation in all three HNSCC cell lines, indicating a reduction in the repair efficiency of DSBs through HR. Whilst not previously shown in HNSCC, a study in pancreatic cancer cells showed pre-treatment with the Chk1 inhibitor AZD7762 resulted in significantly less RAD51 positive cells following irradiation [30], which was further supported by the DR-GFP reporter assay that demonstrated significantly less active HR repair of induced DSBs in the presence of AZD7762. Similarly in pancreatic cancer models, MK-8776 was observed to radiosensitise HR-proficient cell lines, whereas this had no significant influence on BRCA-2 deficient Capan-1 cells, indicating a role for HR in the radiosensitising mechanism [31]. Interestingly, there is some evidence to suggest that the consequences of checkpoint inhibition in combination with radiation are associated with an increase in replication stress [26, 28]. However contradictory to this, and despite the presence of pan-γH2AX staining in HNSCC cells following Wee1 inhibition (MK-1775) pre-treatment, this was determined not to be the major mechanism underlying the increased radiosensitivity [29]. Similarly, we observed inhibition of DNA replication speeds with Chk1 and Wee1 inhibition following X-ray irradiation, although this appeared an additive effect as significant replication stress was observed with the inhibitors alone. Although not explored in our study, there is also evidence that this persistent DNA damage is present in mitotic phases of the cell cycle and therefore induces chromosomal aberrations [10, 20].

In addition to demonstrating the ability of Chk1 and Wee1 inhibition to enhance the radiosensitivity of HNSCC cells towards X-rays, we also show that this strategy can increase the efficacy of PBT at both low and relatively high-LET relative to the Bragg peak. To our knowledge, only two other studies have demonstrated the potential for Chk1 inhibition in combination with PBT, but which were low-LET focussed only. Both studies, one performed in TNBC and the other in pancreatic tumour models, demonstrated an increase in radiosensitivity following pre-treatment with the Chk1 inhibitors PF-00477736 or LY2606368 [14, 32], although the development of PF-00477736 has since been discontinued [33, 34]. Nevertheless, our study using an alternative and more selective Chk1 inhibitor, MK-8776, now provides further evidence of the radiosensitising potential of this strategy following PBT across the Bragg peak where increasing LET is observed. Additionally, we demonstrate that Wee1 inhibition (via MK-1775) can also enhance sensitivity of HNSCC cells and organoids to both low- and high-LET PBT. There are currently no other studies to demonstrate the radiosensitising potential of MK-1775, or any other Wee1 inhibitors, in combination with PBT.

In terms of the underlying mechanisms contributing to the radiosensitisation of HNSCC cells to PBT in the presence of MK-8776 or MK-1775, this does not appear to differ from that in response to X-ray radiation. Indeed, our data generated from γH2AX foci analysis and neutral comet assays demonstrate an increase in the levels and persistence of DSBs post-irradiation, with evidence also that there is a lack of ability to perform efficient HR repair of the damage, specifically following high-LET PBT. Interestingly, our data indicates a reduced overall HR response to low-LET PBT, compared to X-rays and high-LET PBT, and therefore the impact of checkpoint inhibition in HNSCC cells appears minimal under these specific irradiation conditions. Nevertheless, a previous study in TNBC models has demonstrated a reduction in RAD51 activity and increased numbers of γH2AX foci at 24 h post-irradiation with low-LET PBT in combination with the Chk1 inhibitor PF-00477736 [14]. We have also explored the influence of Chk1 and Wee1 inhibition on CDD induced by high-LET PBT using enzyme modified neutral comet assays. This revealed that whilst there was an increased persistence in DNA damage compared to DMSO controls, there seemed to be no greater difference compared to the neutral comet assay in the absence of enzyme modification which reveals DSBs, indicating that DNA damage persistence is largely due to unrepaired DSBs rather than any direct influence on CDD repair, at these time points. Noteworthy is that we have previously demonstrated under similar proton irradiation conditions that the CDD formed is largely non-DSB associated [35]. Whilst we are unaware of any other studies investigating the influence of MK-8776 or MK-1775 in combination with high-LET PBT in HNSCC or any other tumour types, one study has been performed utilising the Chk1 inhibitor UCN-01 in combination with carbon ions, which have considerably higher LET than PBT. A radiosensitisation effect was observed following both X-ray and carbon ion irradiation (184 keV/µm) in HNSCC stem-like cells [36]. Furthermore, two other studies have been performed in non-small cell lung cancer models with carbon ions. Utilising the Chk1 inhibitor AZD7762, it was shown that there was an increase in the persistence of γH2AX foci 24 h post-irradiation with carbon ions in H1299 cells compared to the irradiation alone [37]. In addition, a study investigating the radiosensitising potential of the Wee1 inhibitor MK-1775 with carbon ions also demonstrated an increase in γH2AX intensity in H1299 cells 24 h after the combinatorial treatment [38].

We extended our observations acquired in 2D monolayer cells, to also demonstrate that cell cycle checkpoint inhibition in combination with X-rays or PBT is effective in supressing the growth and viability of 3D spheroids and patient-derived organoids of HNSCC. To our knowledge, only one other study has previously confirmed enhanced radiosensitivity of 3D tumour models to Chk1 inhibition [32]. This study demonstrated the radiosensitising potential of Chk1 inhibition (LY2606368) in 3D pancreatic models (Colo357 and MiaPaCa-2), utilising both X-rays and low-LET PBT (3.7 keV/µm). Chk1 inhibition was shown to increase radiosensitisation to both X-ray and low-LET PBT with DER values of 1.5–1.6 and 1.3, respectively [32]. The use of 3D models, particularly patient-derived organoids, is important as these are considered more appropriate experimental models that accurately reflect the structure of the original tumour and how this responds to treatment. However, the next stage would therefore be to expand on these studies using a larger cohort of models, which would help to understand whether there is any heterogeneity in the radiosensitivity response and potentially the key genetic factors that are driving this. Additionally, it is important to understand the therapeutic ratio of the combinatorial treatment using appropriate normal tissue models (such as pair-matched normal and tumour patient-derived organoids). Beyond this, it is important that experiments employing in vivo (mouse) models using HNSCC tumours are used to further show that MK-8776/MK-1775 in combination with X-rays or PBT can radiosensitise and supress tumour growth. There is some evidence to suggest that the combination of X-rays with Chk1 inhibition, utilising either CCT244747, SAR020106 or LY2606368, can inhibit tumour growth with no measurable toxicity in HNSCC xenograft mouse models [11, 25, 26, 39]. However, there is still need for additional studies utilising both MK-8776 and MK-1775 to explore their potential in HNSCC in vivo models. Nevertheless, our study now provides evidence to support that MK-8776 and MK-1775 are effective radiosensitisers for HNSCC in response to both X-rays and PBT, and which could provide clinical benefits for HNSCC patients.

## Supplementary information

Supplementary Data

## Data Availability

Source data are provided within this paper. Any other data will be made available from the corresponding author upon reasonable request.
