# Supplementary material for: Targeting Chk1 and Wee1 kinases enhances radiosensitivity of 2D and 3D head and neck cancer models to X-rays and low/high-LET protons
Source: Cell Death Dis. 2025 Feb 25;16(1):128. doi: 10.1038/s41419-025-07435-0 (PMC11850709; doi:10.1038/s41419-025-07435-0)
Supplement: Supplementary file 1 — Supplementary Data [file 41419_2025_7435_MOESM1_ESM.docx]

**SUPPLEMENTARY DATA**


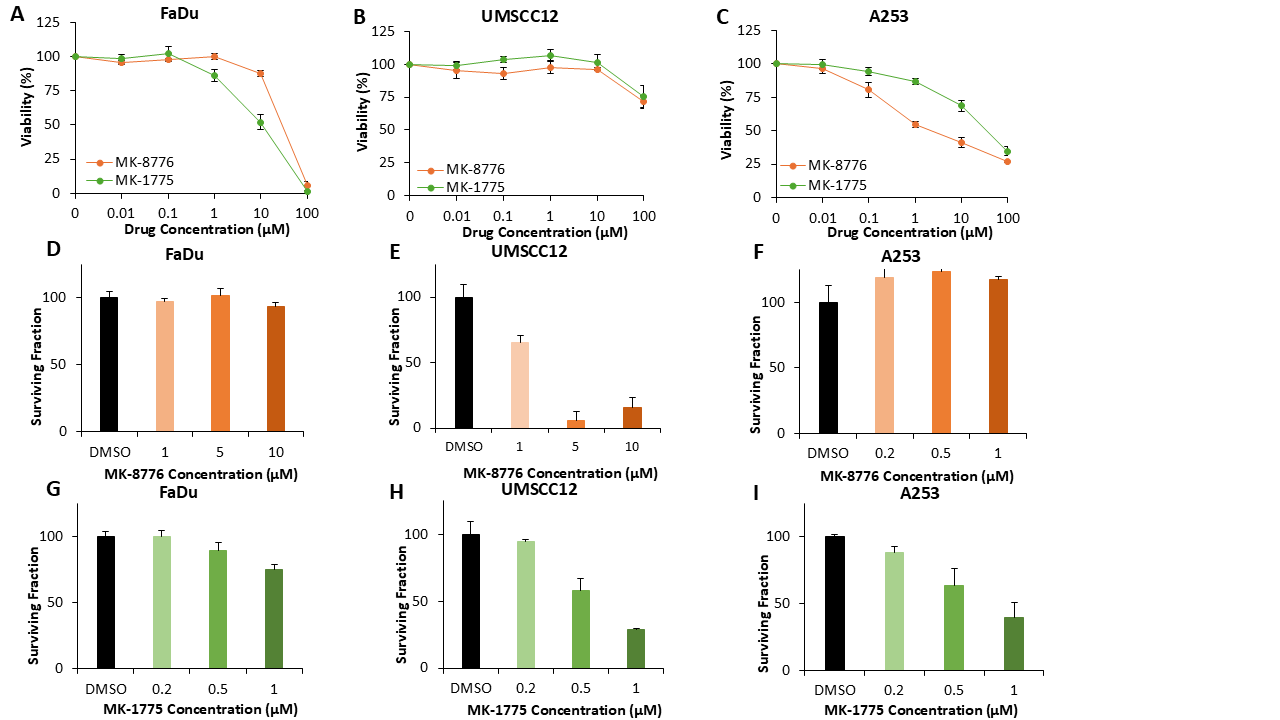


**Supplementary Figure 1. Viability and survival of HNSCC cells in response to MK-8776 and MK-1775.** (A) FaDu, (B) UMSCC12 and (C) A253 monolayer cells were treated with increasing doses of either MK-8776 or MK-1775 for 72 h. Cell viability was measured via CellTiter Blue from three biologically independent experiments. Shown is the mean cellular survival±SE. (D) FaDu, (E) UMSCC12 and (F) A253 were treated as single cells with MK-8776 (from 0.2-10 μM) for ~16 h. (G) FaDu, (H), UMSCC12 and (I) A253 were treated as single cells with MK-1775 (0.2-1 μM) for ~16 h. Cells were left to form colonies, stained and counted to determine HNSCC survival. Shown is the mean surviving fraction±SE.

**
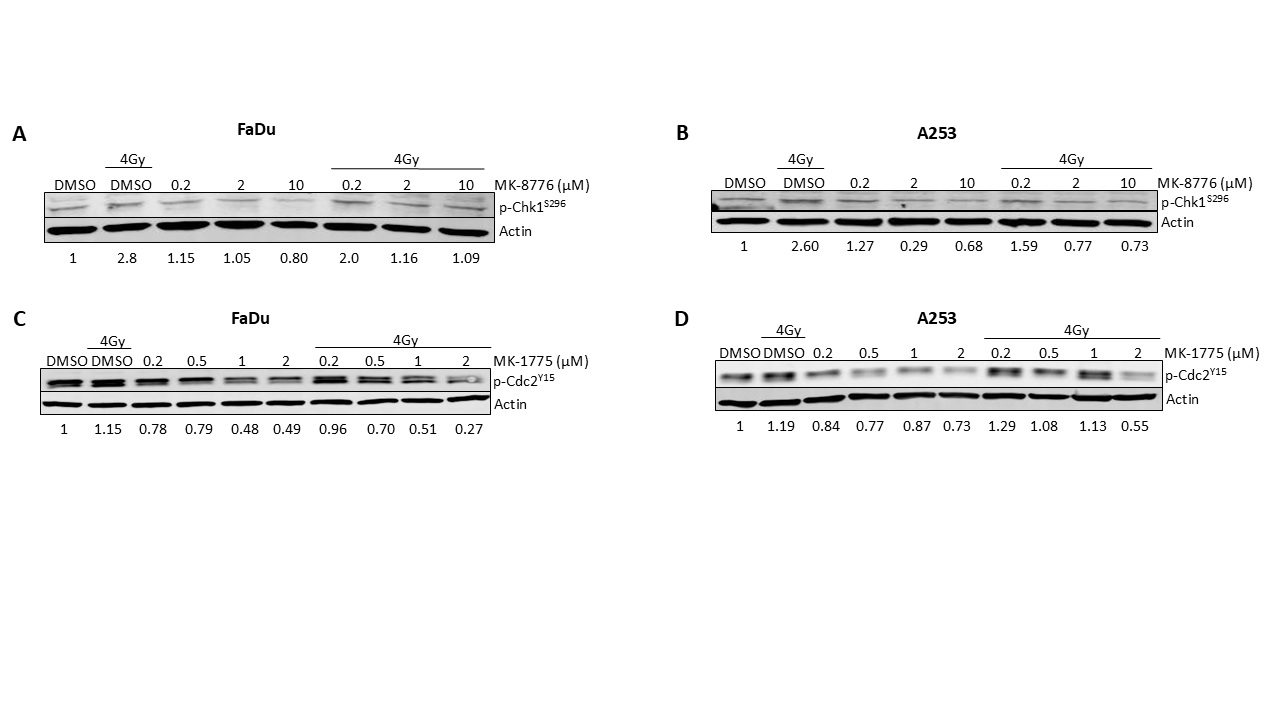
**

**Supplementary Figure 2. MK-8776 and MK-1775 effectively inhibit Chk1 and Wee1 kinases in HNSCC cells.** (A, C) FaDu and (B, D) A253 cells were pre-treated with various doses of either MK-8776, MK-1775 or DMSO for ~16 h prior to exposure to 4 Gy X-Rays, or in the absence of irradiation. Whole cell extracts were prepared and the levels of autophosphorylation of Chk1 at phospho-site S296 or phosphorylation of the downstream target Cdc2 at phospho-site Y15 were measured using immunoblotting. Quantification shown is normalised to the unirradiated DMSO controls.

**
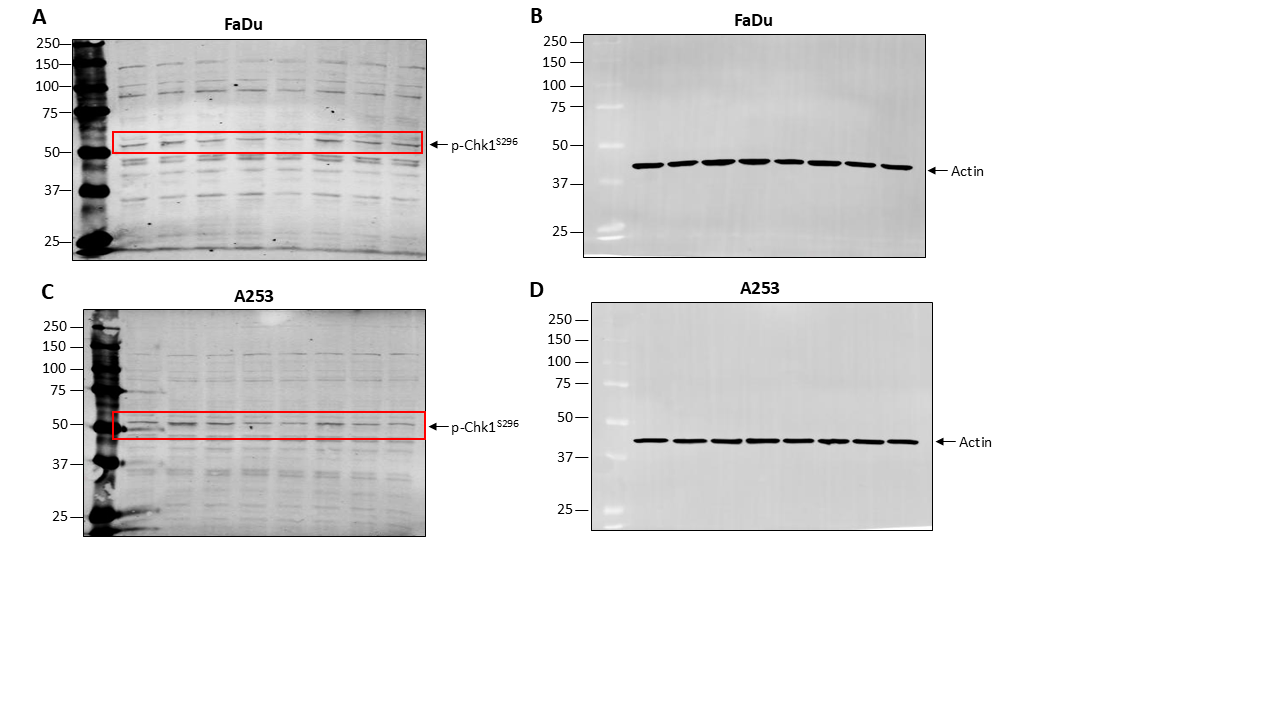
**

**Supplementary Figure 3. Full length blots for Supplementary Figure 2A-B.** (A) FaDu cells showing Chk1 phospho-site serine 296 (S296) and (B) actin as loading control. (C) A253 cells showing Chk1 phospho-site serine 296 (S296) and (D) actin as a loading control.

**
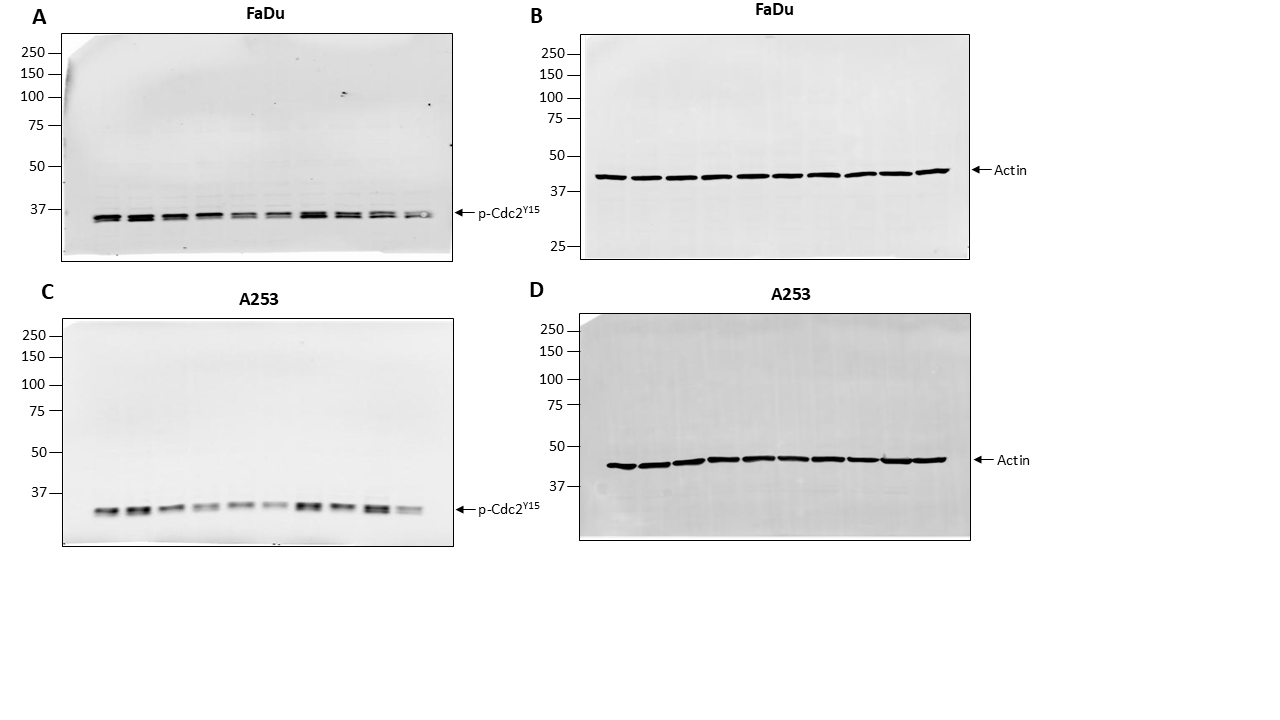
**

**Supplementary Figure 4. Full length blots for Supplementary Figure 2C-D.** (A) FaDu cells showing Cdc2 phospho-site tyrosine 15 (Y15) and (B) actin as loading control. (C) A253 cells showing Cdc2 phospho-site tyrosine 15 (Y15) and (D) actin as a loading control.

**
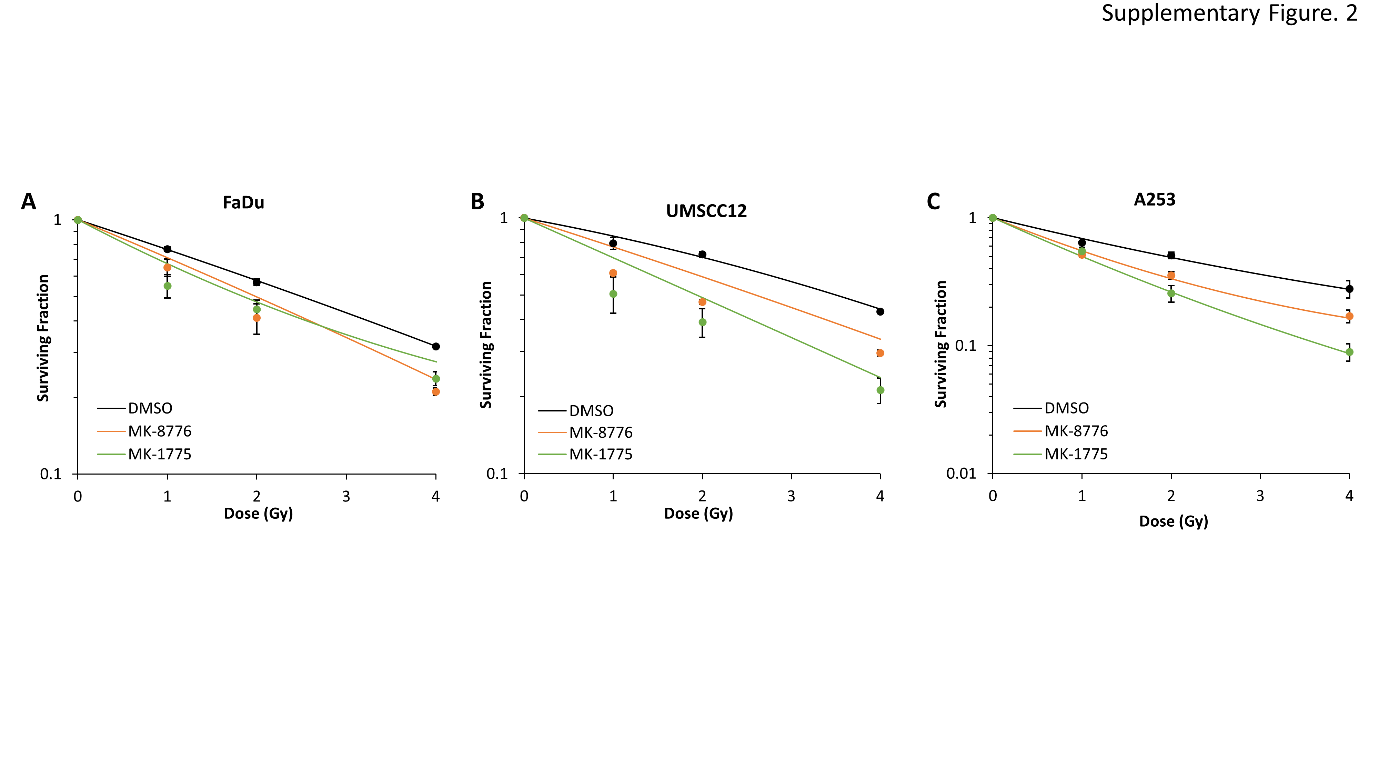
**

**Supplementary Figure 5. Inhibition of Chk1 or Wee1 results in increased radiosensitivity of HNSCC cells to X-ray radiation.** (A) FaDu, (B) UMSCC-12 or (C) A253 cells were treated with either 1 μM MK-8776 (10 μM for FaDu) or 0.2 μM MK-1775 for ~16 h prior to exposure to X-ray radiation, and clonogenic survival of the cells was analysed from three biologically independent experiments. Shown is the data fitted according to the linear quadratic (LQ) model.

**
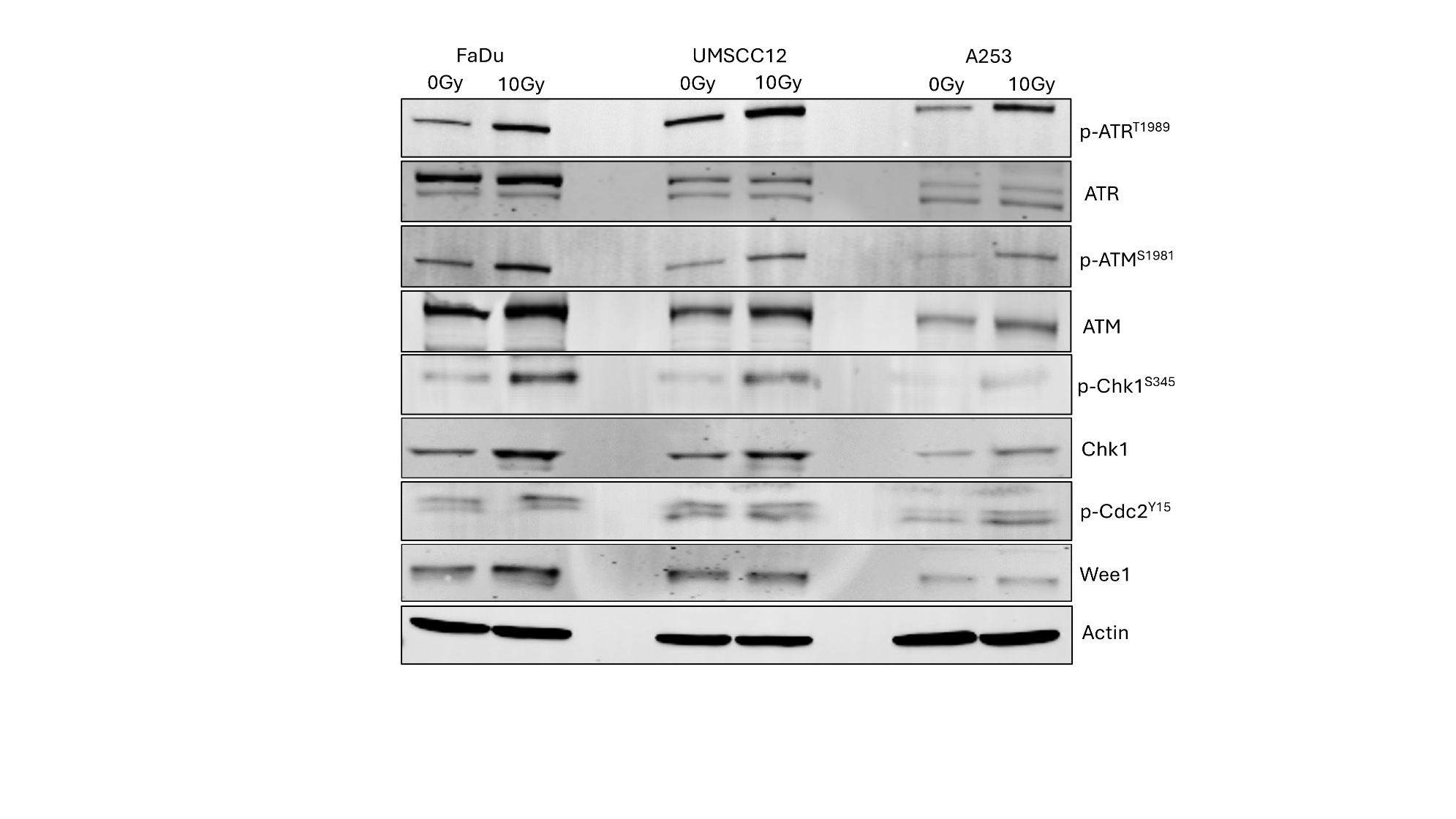
**

**Supplementary Figure 6. Protein levels of key DNA damage repair and cell cycle checkpoint proteins in HNSCC cell lines pre and post X-ray radiation.** FaDu, UMSCC12 and A253 cell lines were either unirradiated, or irradiated with 10 Gy X-ray radiation and cells harvested 4 h post-irradiation. Whole cell extracts were prepared, and proteins analysed using the indicated antibodies by immunoblotting.


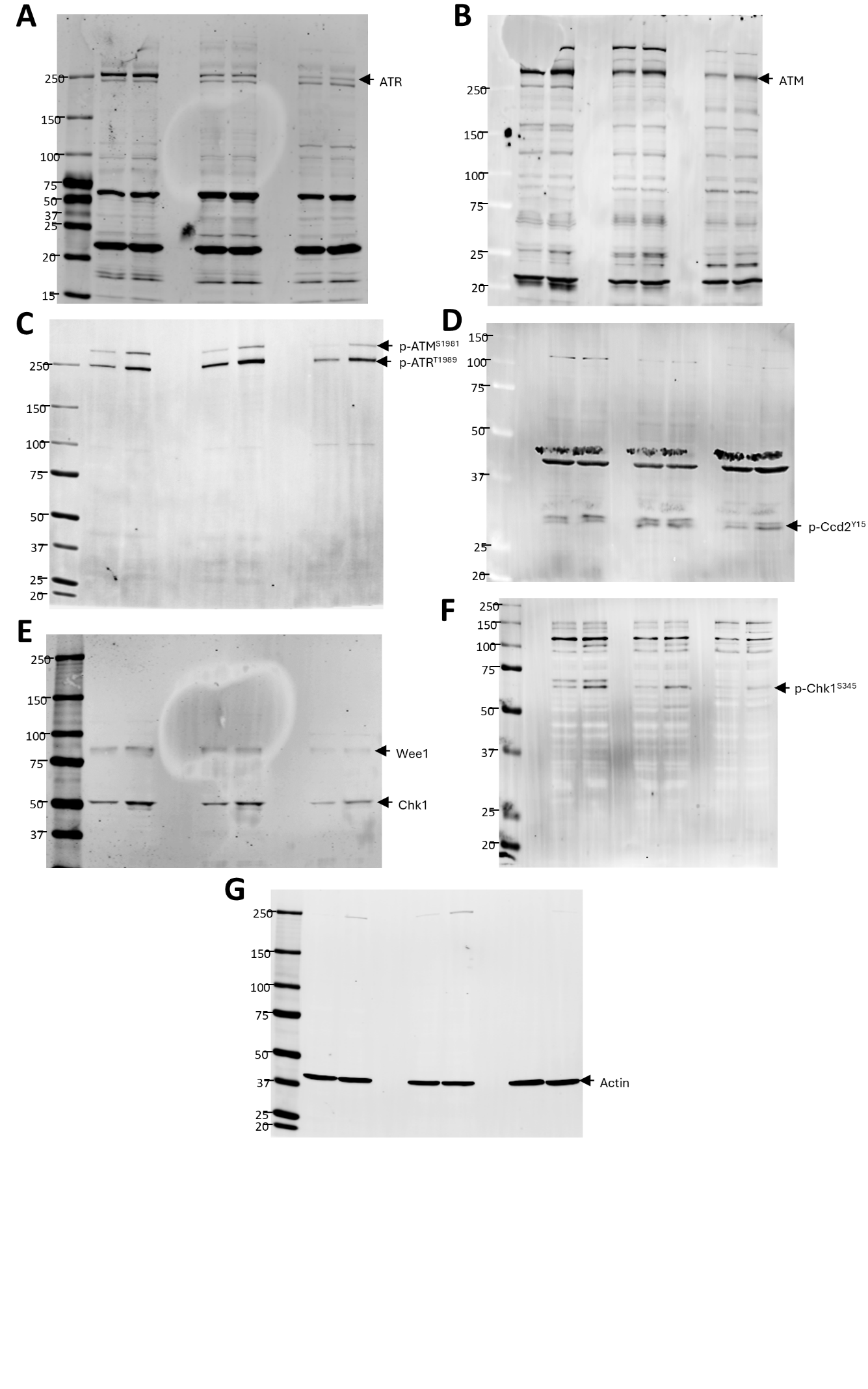


**Supplementary Figure 7. Full length blots for Supplementary Figure 6A.** Proteins analysed from HNSCC cells showing (A) ATR, (B) ATM, (C) ATM phospho-site serine 1981 (S1981) and ATR phospho-site threonine 1989 (T1989), (D) Cdc2 phospho-site tyrosine 15 (Y15), (E) Chk1 and Wee1, (F) Chk1 phospho-site serine 345 (S345) and (G) actin as a loading control.

**
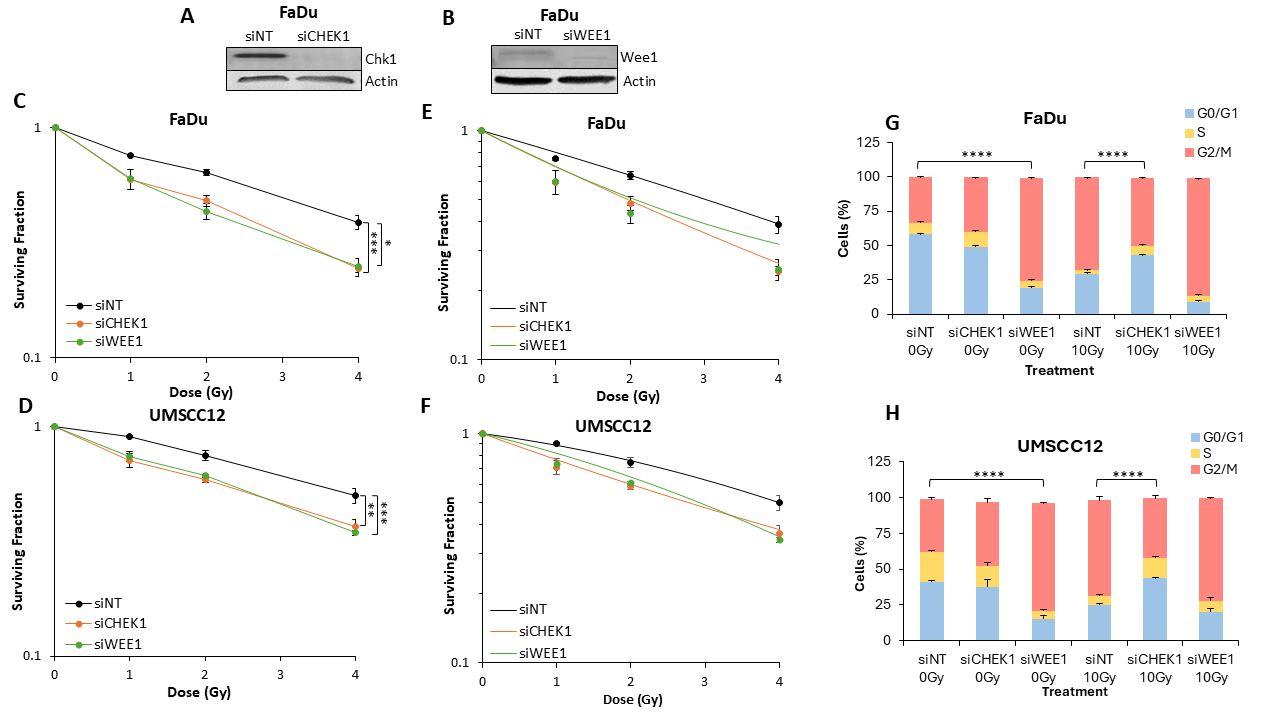
**

**Supplementary Figure 8. siRNA knockdown of Chk1 or Wee1 radiosensitises HNSCC cells to X-ray radiation.** FaDu cells were treated with siRNA targeting either (A) Chk1 (siCHEK1), (B) Wee1 (siWEE1) or non-targeting siRNA (siNT) for 48 h. Whole cell extracts were then prepared and immunoblotting performed with the indicated antibodies. Alternatively, and following siRNA treatment, (C, E) FaDu or (D, F) UMSCC12 cells were irradiated with X-rays and clonogenic survival of cells was analysed from three biologically independent experiments. (C-D) Shown is the mean surviving fraction±SE. *p<0.02, **p<0.001, ***p<0.0001, as calculated using the CFAssay for R. (E-F) Data has been fitted according to the linear quadratic (LQ) model. (G) FaDu and (H) UMSCC12 cells pre-treated with siRNA for either CHEK1 or WEE1 for 48 h prior to exposure to 10 Gy X-ray radiation and fixed 24 h later. Cells were then analysed for cell cycle phases using flow cytometry. Shown is the mean % cells±SE. Statistical analysis was performed using a one-sample *t* test. ****p<0.00001.

**
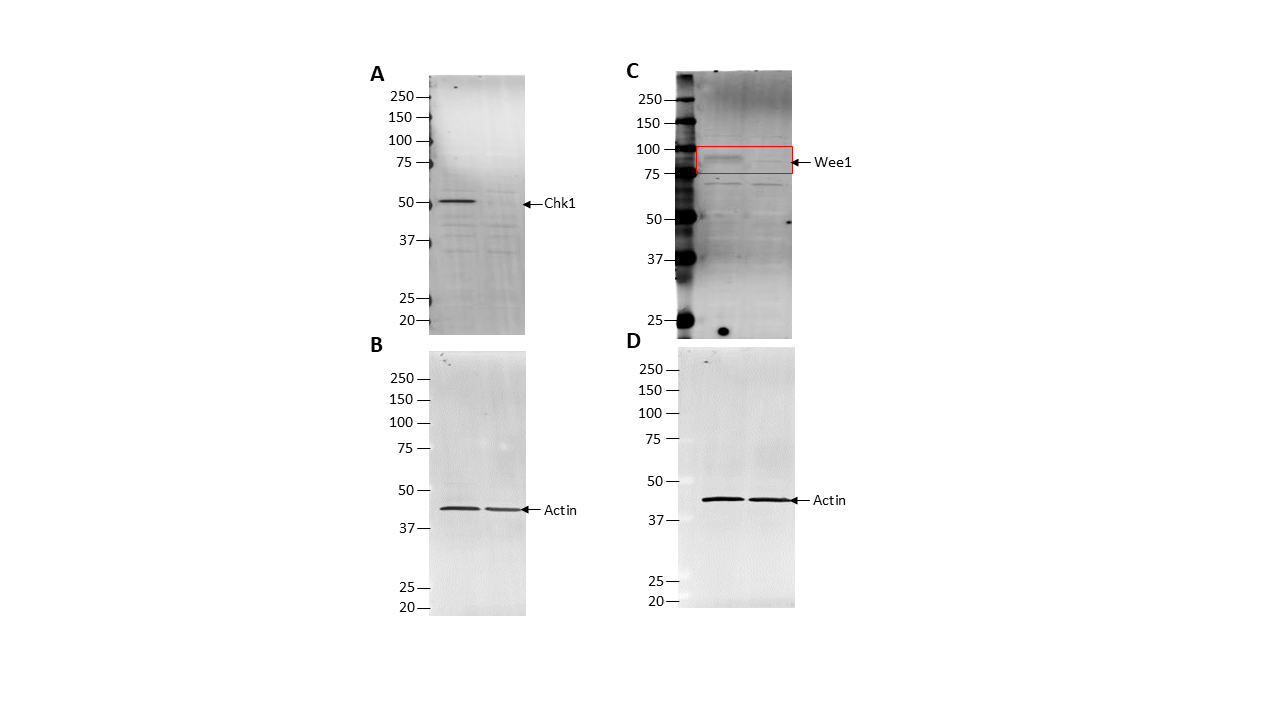
**

**Supplementary Figure 9. Full length blots for Supplementary Figure 8A-B.** (A) Chk1, (C) Wee1 and (B, D) actin as a loading control from FaDu cells treated with CHEK1 and WEE1 siRNA.

**
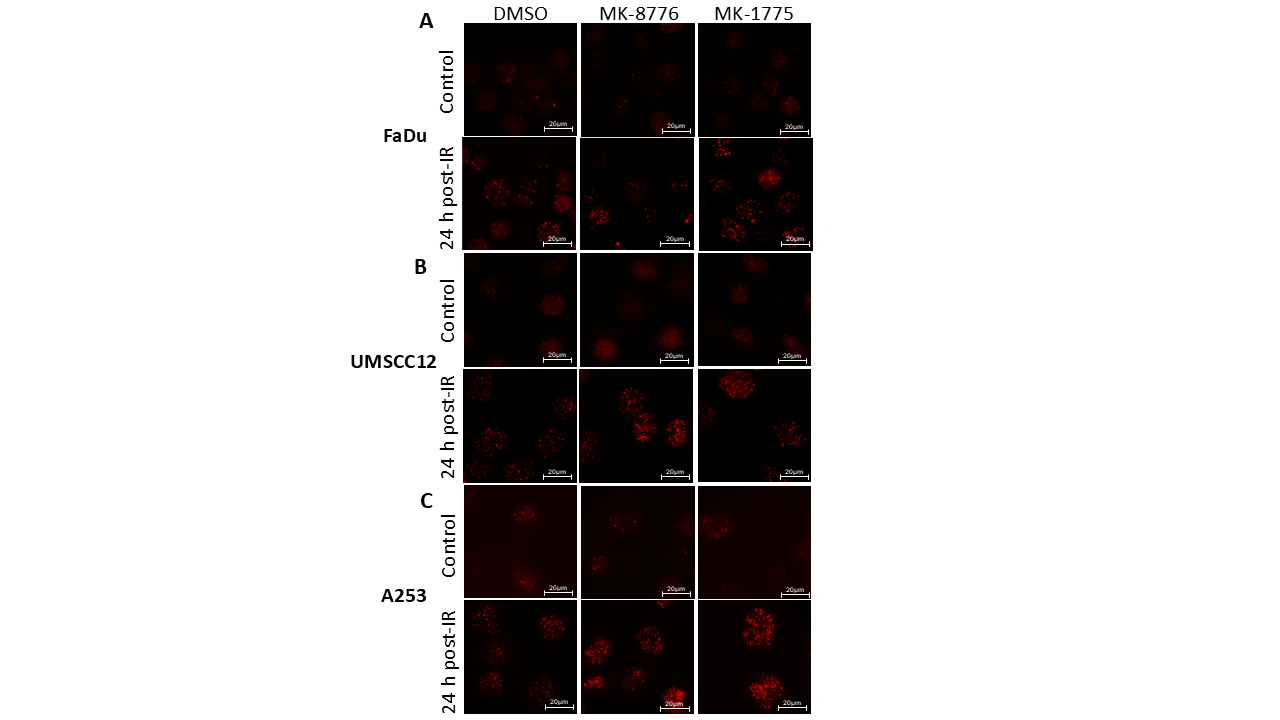
**

**Supplementary Figure 10. Chk1 or Wee1 inhibition causes increased persistence of X-ray radiation-induced DSBs.** (A) FaDu, (B) UMSCC12 or (C) A253 cells were treated with 1 μM MK-8776 (10 μM for FaDu) or 0.2 μM MK-1775 for ~16 h prior to exposure to 4 Gy X-ray radiation. DNA DSB damage was measured at various timepoints post-irradiation using γH2AX foci through immunofluorescence microscopy. Shown are representative images for the unirradiated controls, plus 24 h post-irradiation. Scale bar is 20 μm.

**
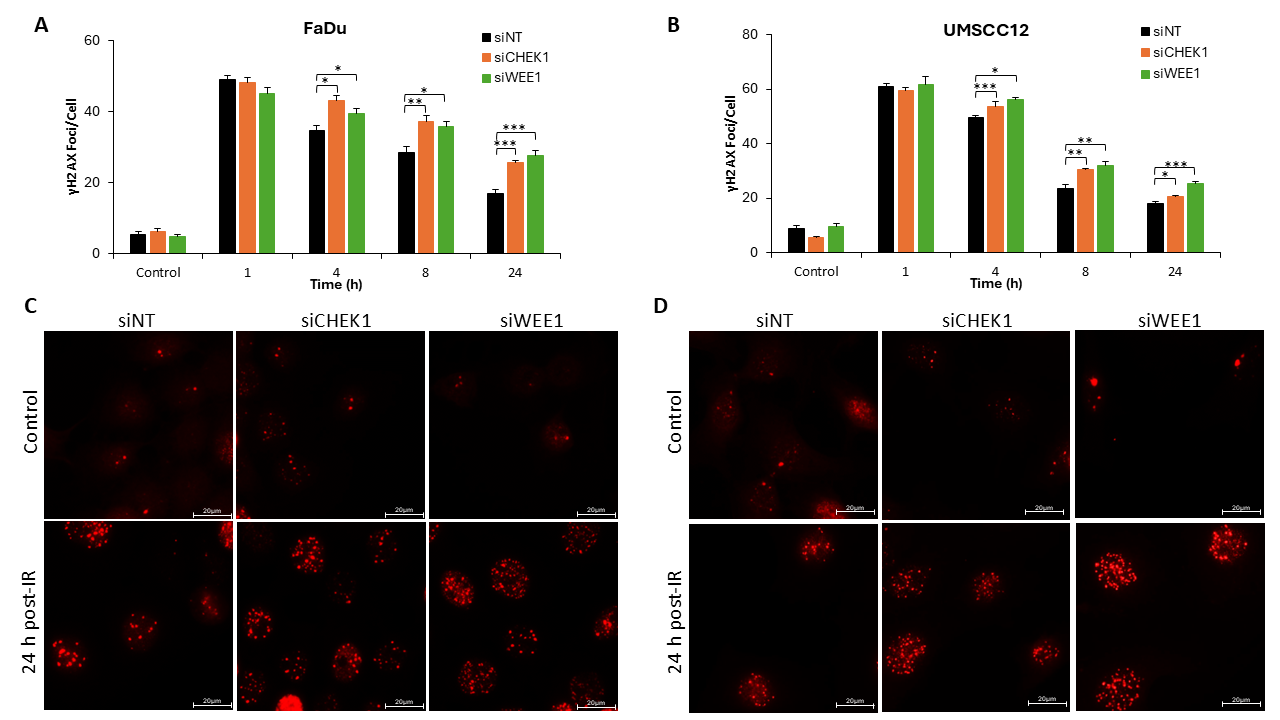
**

**Supplementary Figure 11. siRNA knockdown of either CHEK1 or WEE1 increases persistence of DNA damage post-X-ray irradiation.** (A) FaDu and (B) UMSCC12 cell lines were treated with siRNA for either CHEK1 or WEE1 for 48 h prior to exposure to 4 Gy X-ray radiation. DNA DSB damage was measured at various timepoints post-irradiation using γH2AX foci as a marker through immunofluorescence microscopy. (C-D) Representative images of unirradiated controls, plus 24 h post-irradiation. Scale bar is 20 μm.


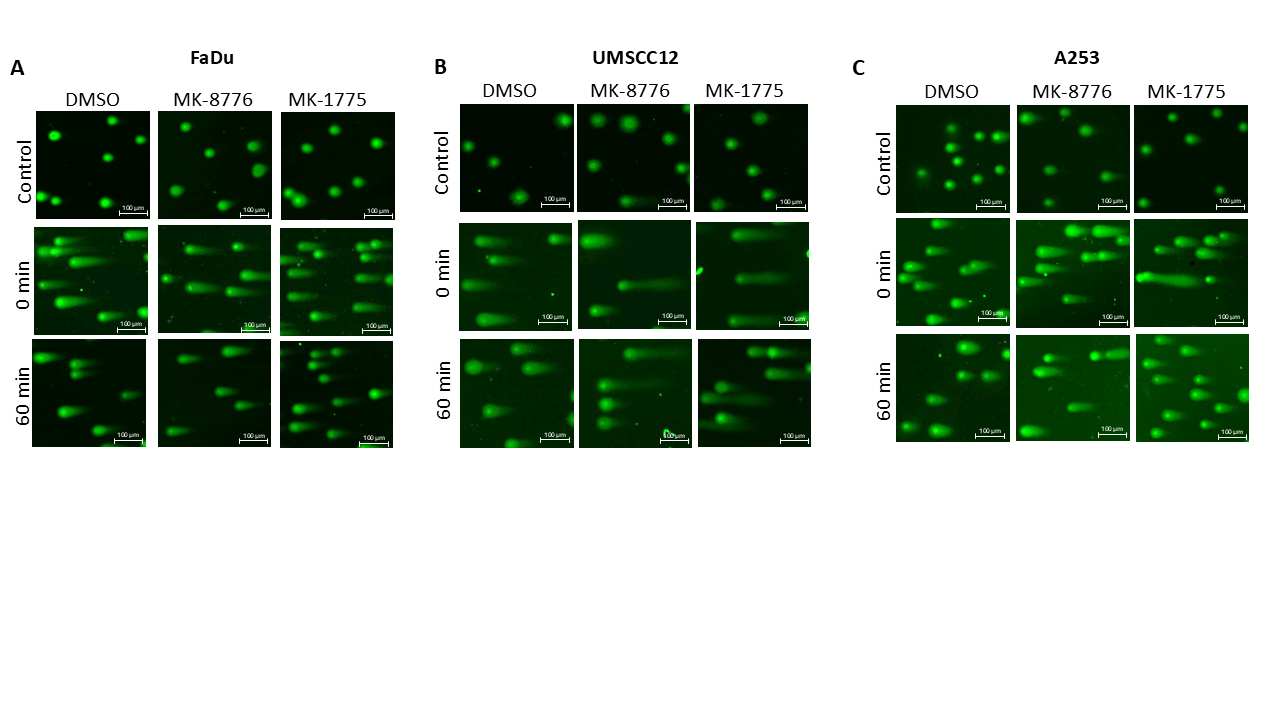


**Supplementary Figure 12. Chk1 or Wee1 inhibition causes increased persistence of X-ray radiation-induced DSBs.** (A) FaDu, (B) UMSCC12 or (C) A253 cells were treated with 1 μM MK-8776 (10 μM for FaDu) or 0.2 μM MK-1775 for ~16 h prior to exposure to 4 Gy X-ray radiation. DNA DSB damage was measured at various timepoints post-irradiation using the neutral comet assay. Shown are representative images for the unirradiated controls, 0 and 60 min post-irradiation. Scale bar is 100 μm.


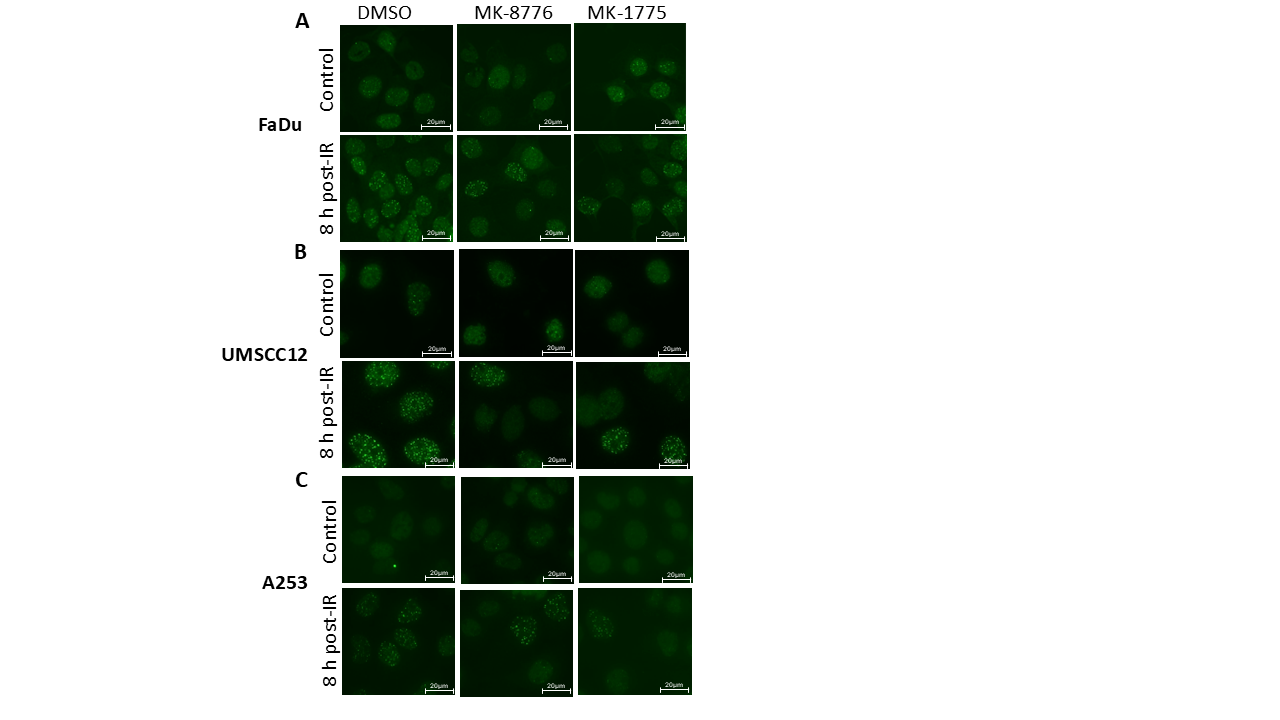


**Supplementary Figure 13. Chk1 or Wee1 inhibition causes increased persistence of X-ray radiation-induced DSBs.** (A) FaDu, (B) UMSCC12 or (C) A253 cells were treated with 1 μM MK-8776 (10 μM for FaDu) or 0.2 μM MK-1775 for ~16 h prior to exposure to 4 Gy X-ray radiation. DNA DSB repair was measured at various timepoints post-irradiation using RAD51 foci through immunofluorescence microscopy. Shown are representative images for the unirradiated controls, plus 8 h post-irradiation. Scale bar is 20 μm.


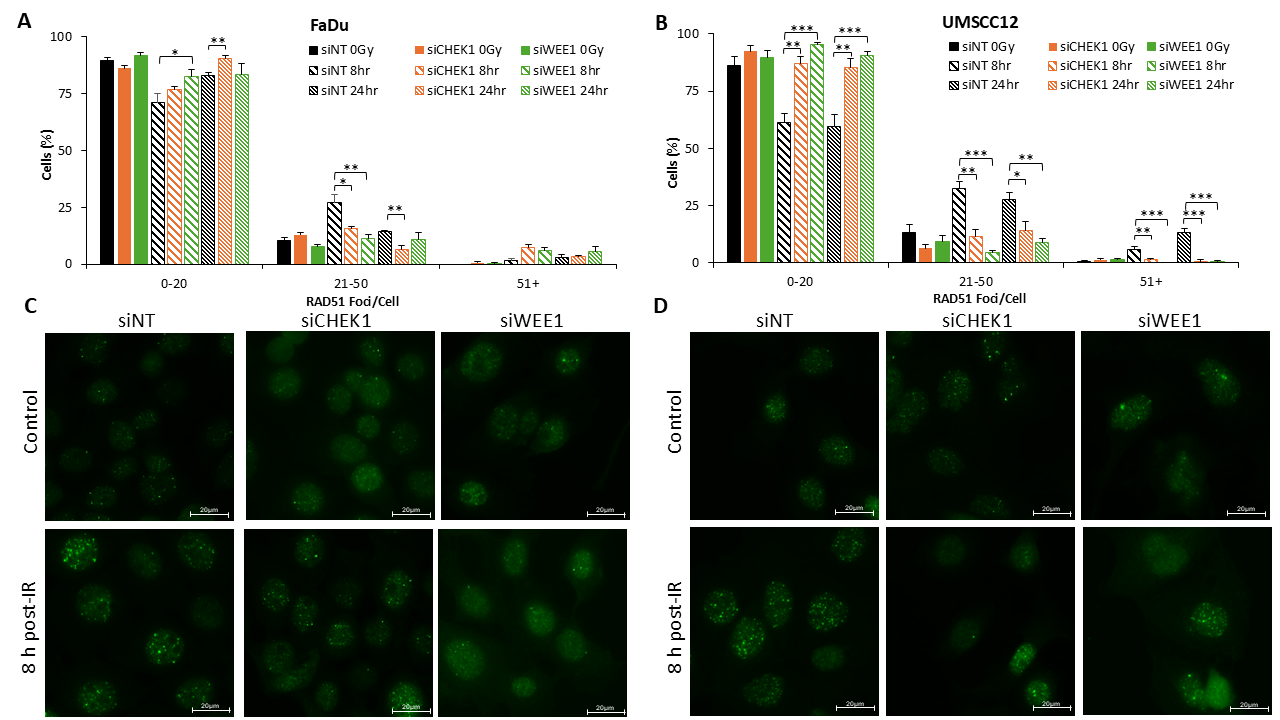


**Supplementary Figure 14. siRNA knockdown of either CHEK1 or WEE1 reduces efficiency of HR repair of DNA DSB damage.** (A) FaDu and (B) UMSCC12 cell lines were treated with siRNA for either CHEK1 or WEE1 for 48 h prior to exposure to 4 Gy X-ray radiation. DNA DSB repair was measured at various timepoints post-irradiation using RAD51 foci as a marker using immunofluorescence microscopy. (C-D) Representative images for unirradiated controls, plus 8 h post-irradiation. Scale bar is 20 μm.

**
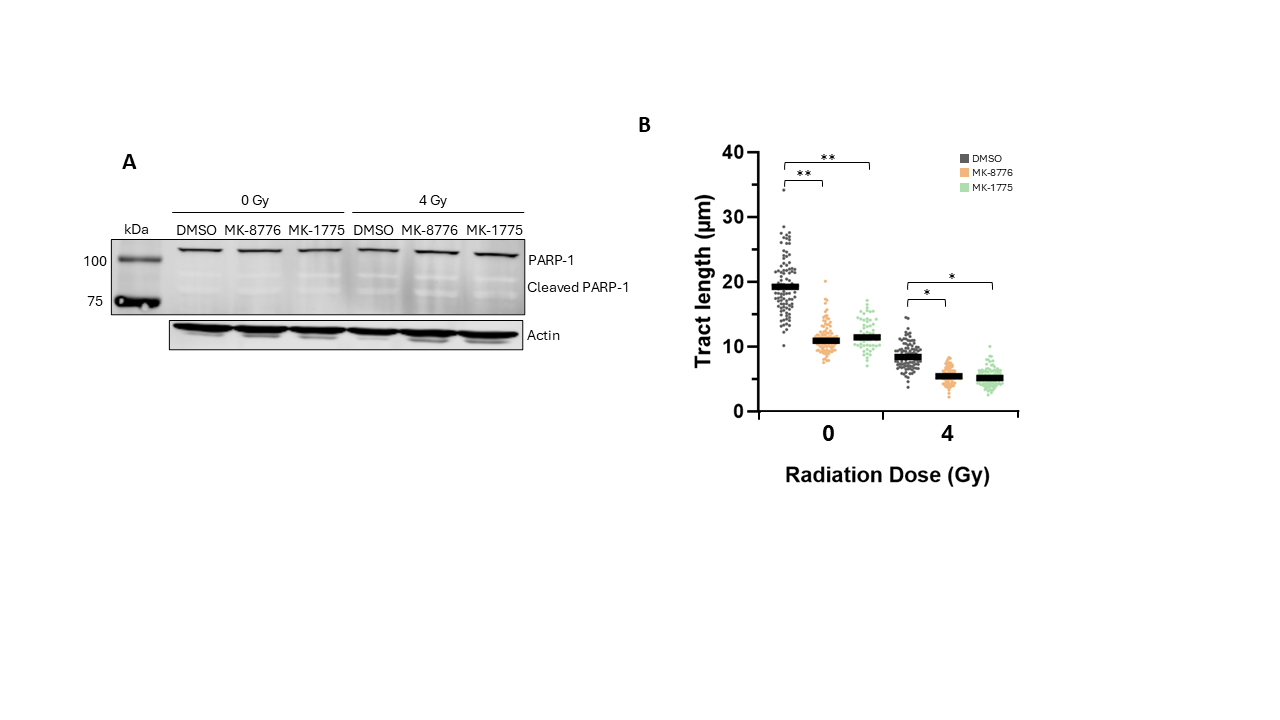
**

**Supplementary Figure 15. Replication stress or apoptosis do not contribute to the mechanism of radiosensitisation following Chk1 or Wee1 inhibition.** (A) FaDu cells were pre-treated with MK-8776 and MK-1775, using DMSO as a control, for ~16 h prior to either 4 Gy X-ray radiation and fixed 24 h later, or left unirradiated. Cells were harvested, whole cell extracts prepared and proteins analysed by immunoblotting using antibodies against PARP-1 (113 kDa), to show PARP-1 cleavage (89 kDa) as a marker of apoptosis, and actin as a loading control. (B) FaDu cells were pre-treated with MK-8776 or MK-1775 for ~16 h before 4 Gy X-ray radiation. The DNA fibre spreading assay was used to visualise and measure DNA tract length, indicating replication speed. Statistical analysis was performed using a Mann-Whitney test, *p<0.001, **p<0.00001.

**
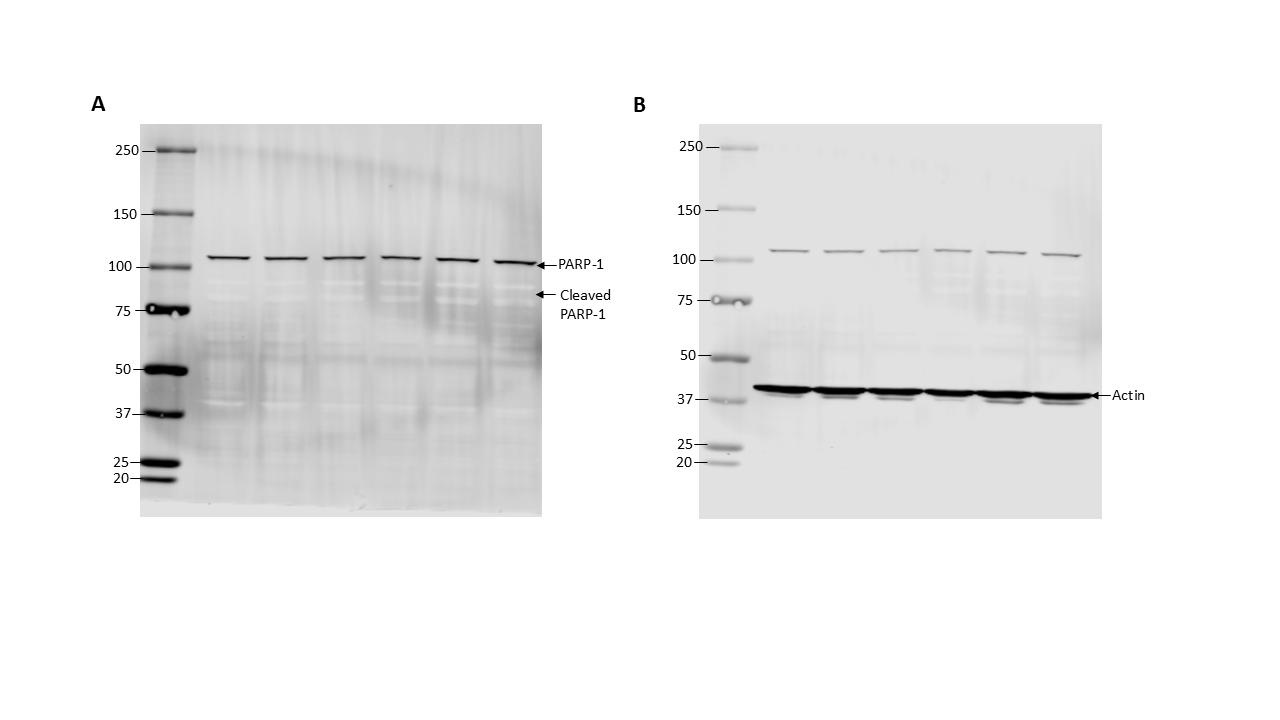
**

**Supplementary Figure 16. Full length blots for Supplementary Figure 16B.** FaDu cells were treated with 10 μM MK-8776 and 0.2 μM MK-1775 for ~16 h prior to 4 Gy X-ray radiation. Cells were harvested 24 h post-irradiation for protein extraction. Immunoblotting for (A) PARP-1 (113 kDa) and cleaved PARP-1 (89 kDa) with (B) actin as a loading control.

**
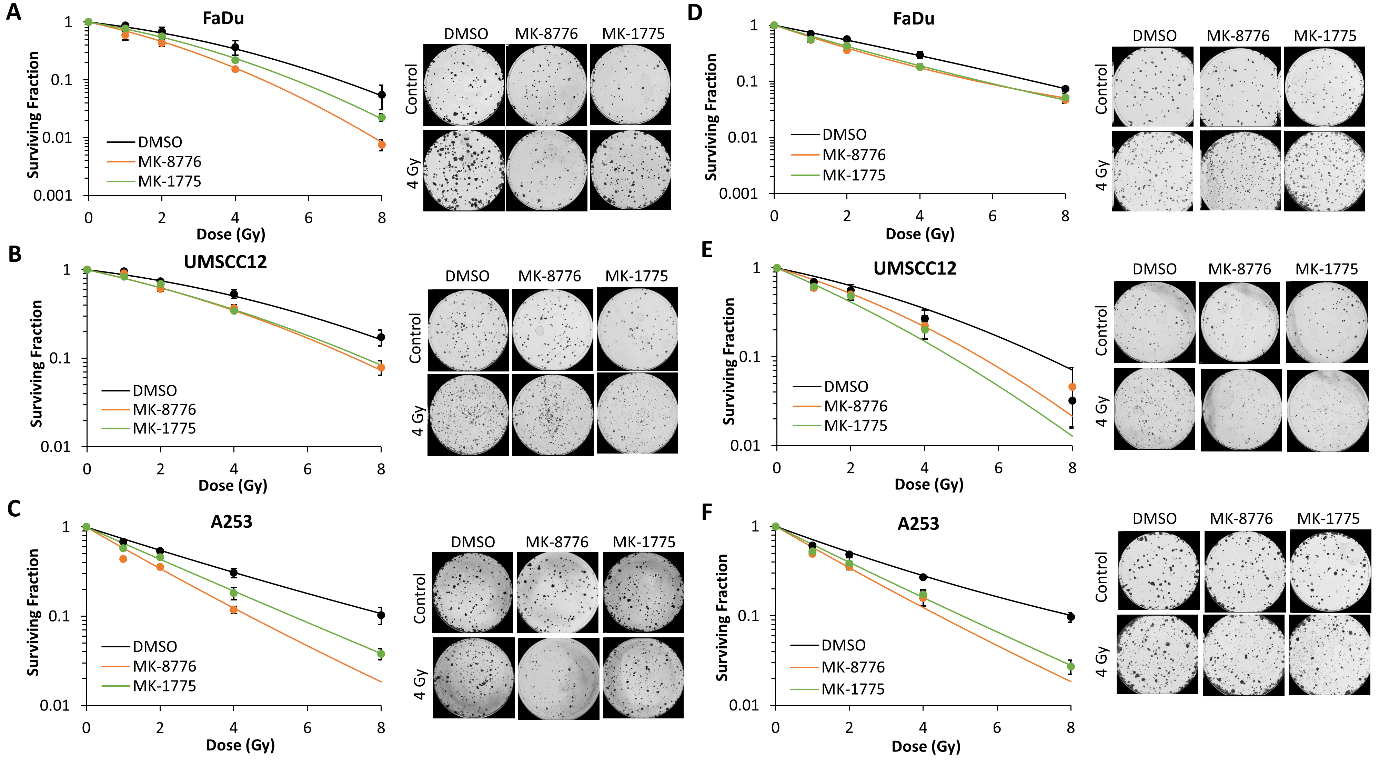
**

**Supplementary Figure 17. Inhibition of Chk1 or Wee1 results in increased radiosensitivity of HNSCC cells to both low and relatively high-LET PBT.** (A, D) FaDu, (B, E) UMSCC12 or (C, F) A253 cells were treated with either 1 μM MK-8776 (10 μM for FaDu) or 0.2 μM MK-1775 for ~16 h prior to exposure to either (A-C) low-LET or (D-F) relatively high-LET PBT. Clonogenic survival of the cells was analysed from three biologically independent experiments, and shown is the data fitted according to the linear quadratic (LQ) model. RBE values calculated at 50 % survival comparing low and high-LET protons are 1.12, 1.28 and 1.42 for A253, FaDu and UMSCC12, respectively. Representative images of colony formation are shown for the unirradiated controls and following 4 Gy X-ray irradiation.

**
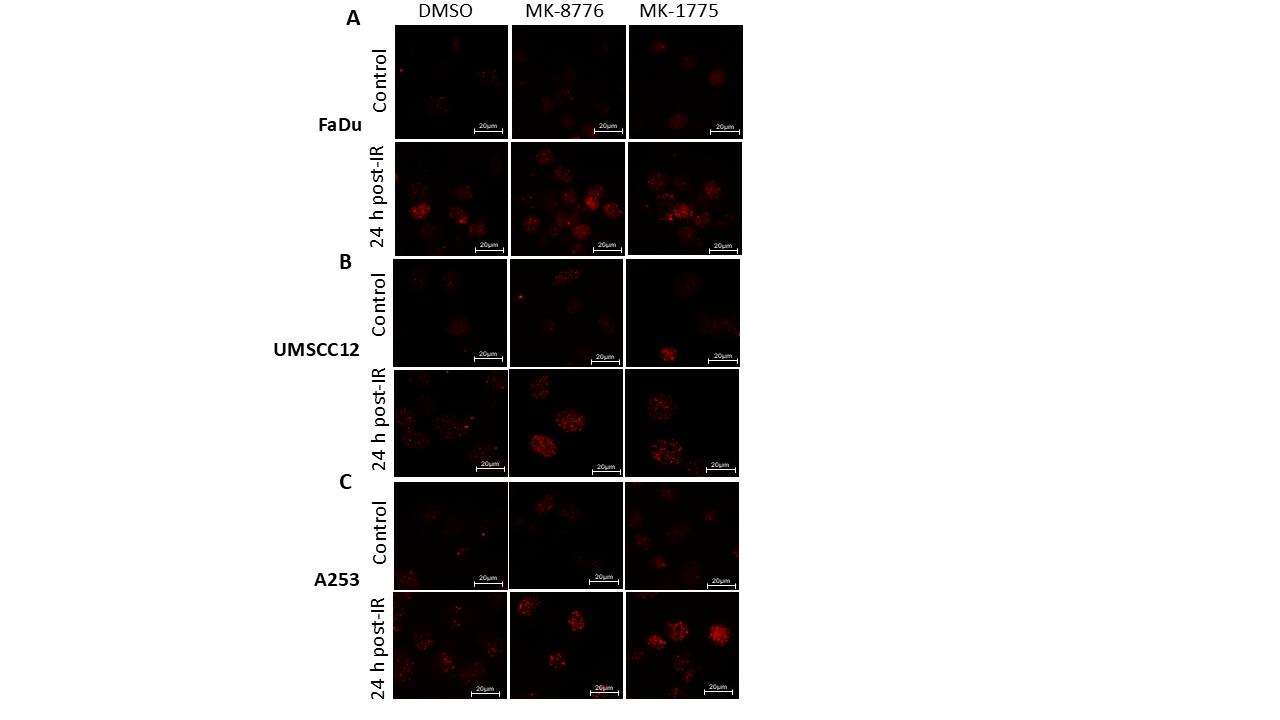
**

**Supplementary Figure 18. Chk1 or Wee1 inhibition causes increased persistence of low-LET PBT-induced DSBs.** (A) FaDu, (B) UMSCC12 or (C) A253 cells were treated with 1 μM MK-8776 (10 μM for FaDu) or 0.2 μM MK-1775 for ~16 h prior to exposure to 4 Gy low-LET PBT. DNA DSB damage was measured at various timepoints post-irradiation using γH2AX foci through immunofluorescence microscopy. Shown are representative images for the unirradiated controls, plus 24 h post-irradiation. Scale bar is 20 μm.

**
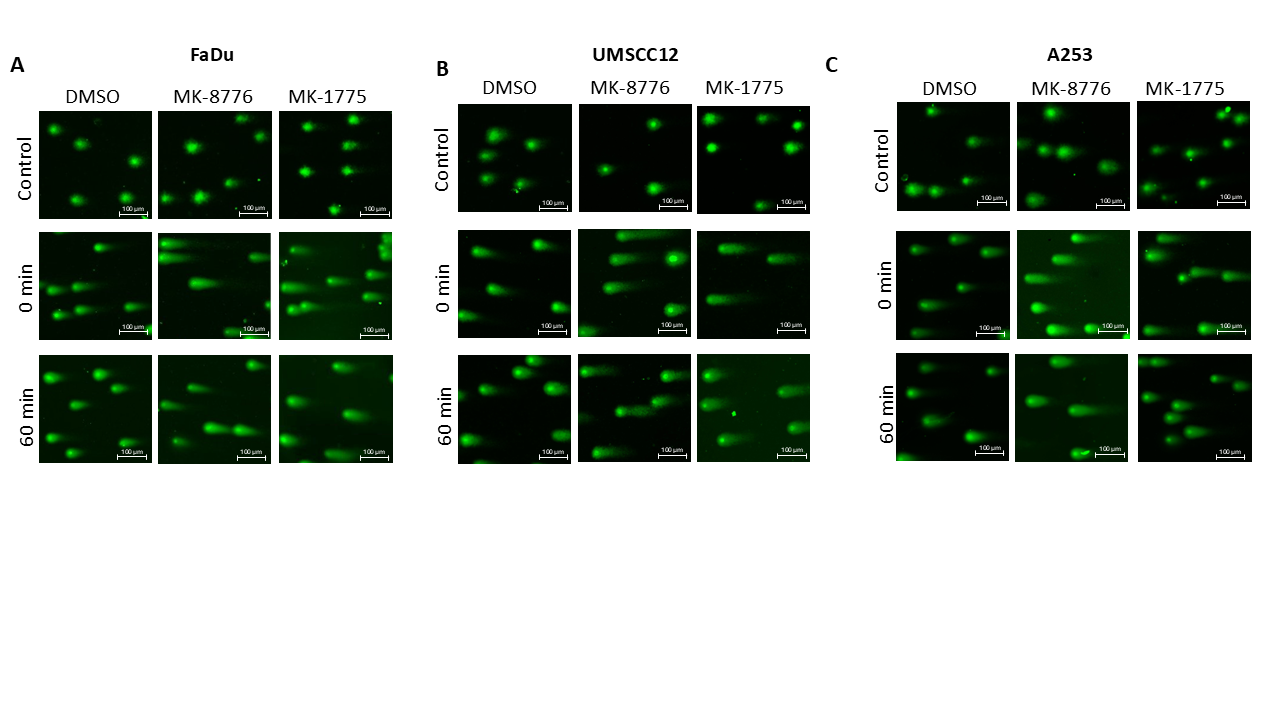
**

**Supplementary Figure 19. Chk1 or Wee1 inhibition causes increased persistence of low-LET PBT-induced DSBs.** (A) FaDu, (B) UMSCC12 or (C) A253 cells were treated with 1 μM MK-8776 (10 μM for FaDu) or 0.2 μM MK-1775 for ~16 h prior to exposure to 4 Gy low-LET PBT. DNA DSB damage was measured at various timepoints post-irradiation using the neutral comet assay. Shown are representative images for the unirradiated controls, 0 min and 60 min post-irradiation. Scale bar is 100 μm.

**
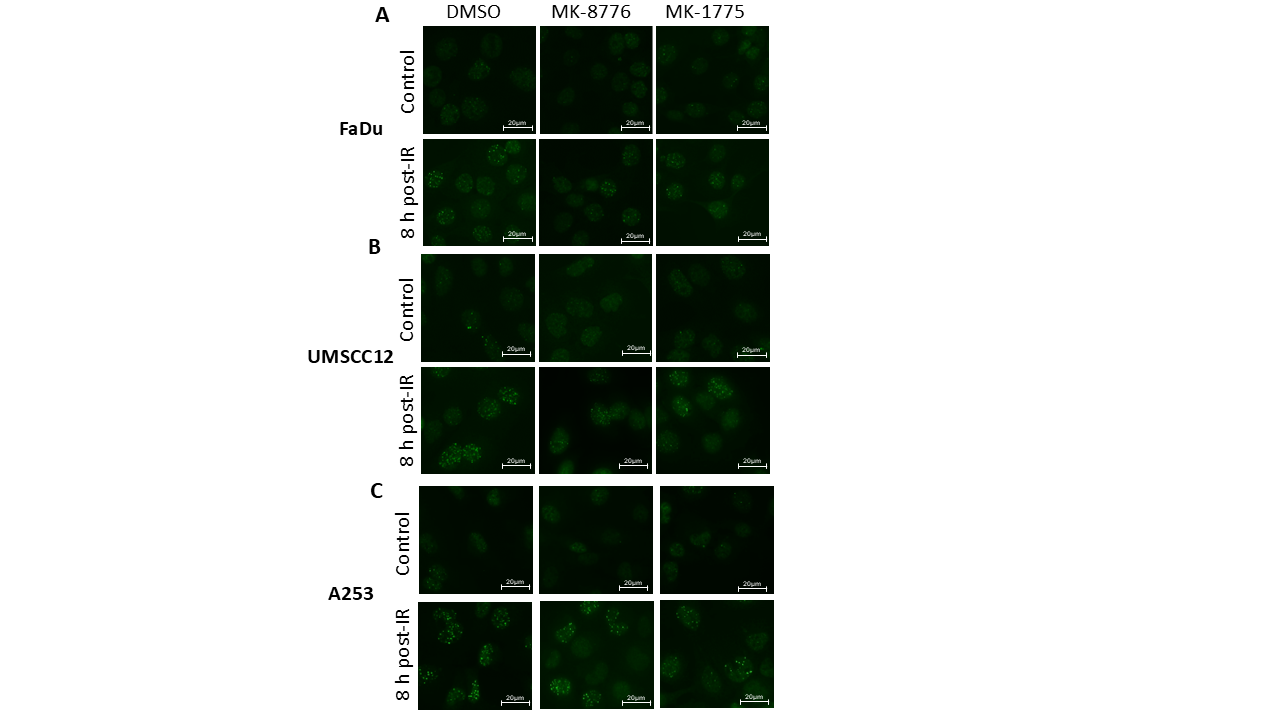
**

**Supplementary Figure 20. Chk1 or Wee1 inhibition causes increased persistence of low-LET PBT-induced DSBs.** (A) FaDu, (B) UMSCC12 or (C) A253 cells were treated with 1 μM MK-8776 (10 μM for FaDu) or 0.2 μM MK-1775 for ~16 h prior to exposure to 4 Gy low-LET PBT. DNA DSB repair was measured at various timepoints post-irradiation using RAD51 foci through immunofluorescence microscopy. Shown are representative images for the unirradiated controls, plus 8 h post-irradiation. Scale bar is 20 μm.


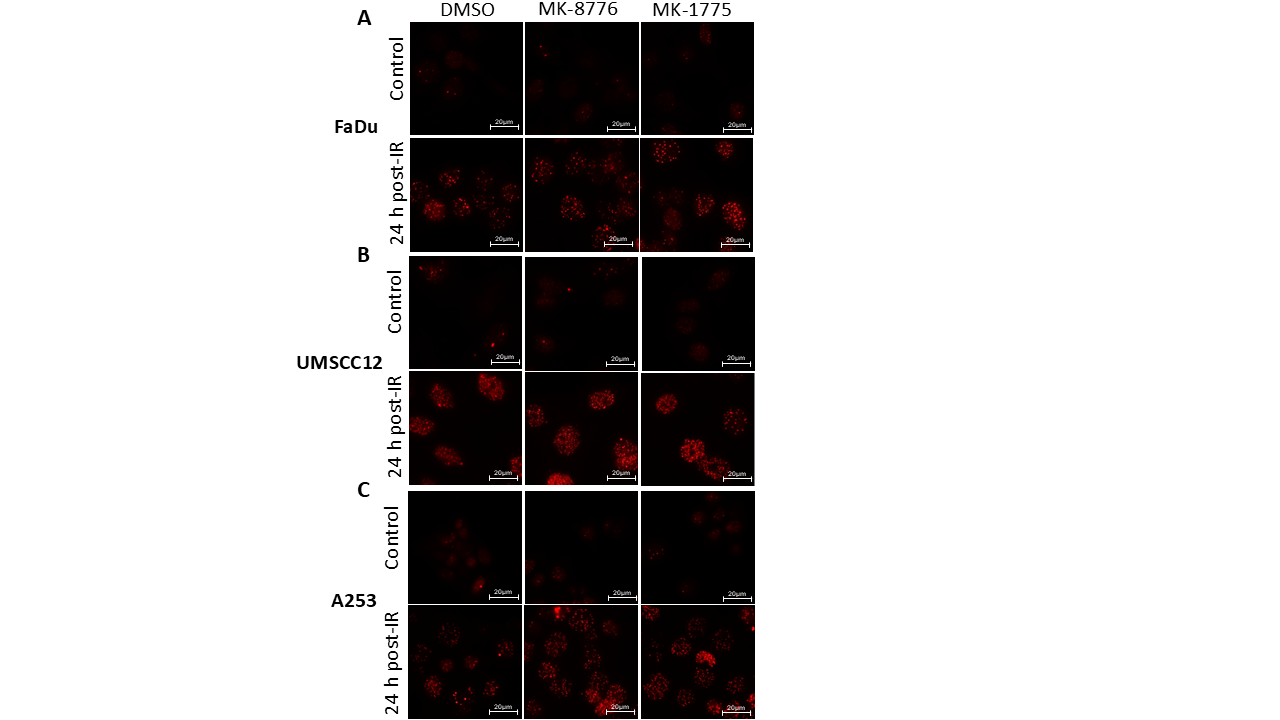


**Supplementary Figure 21. Chk1 or Wee1 inhibition causes increased persistence of high-LET PBT-induced DSBs.** (A) FaDu, (B) UMSCC12 or (C) A253 cells were treated with 1 μM MK-8776 (10 μM for FaDu) or 0.2 μM MK-1775 for ~16 h prior to exposure to 4 Gy high-LET PBT. DNA DSB damage was measured at various timepoints post-irradiation using γH2AX foci through immunofluorescence microscopy. Shown are representative images for the unirradiated controls, plus 24 h post-irradiation. Scale bar is 20 μm.


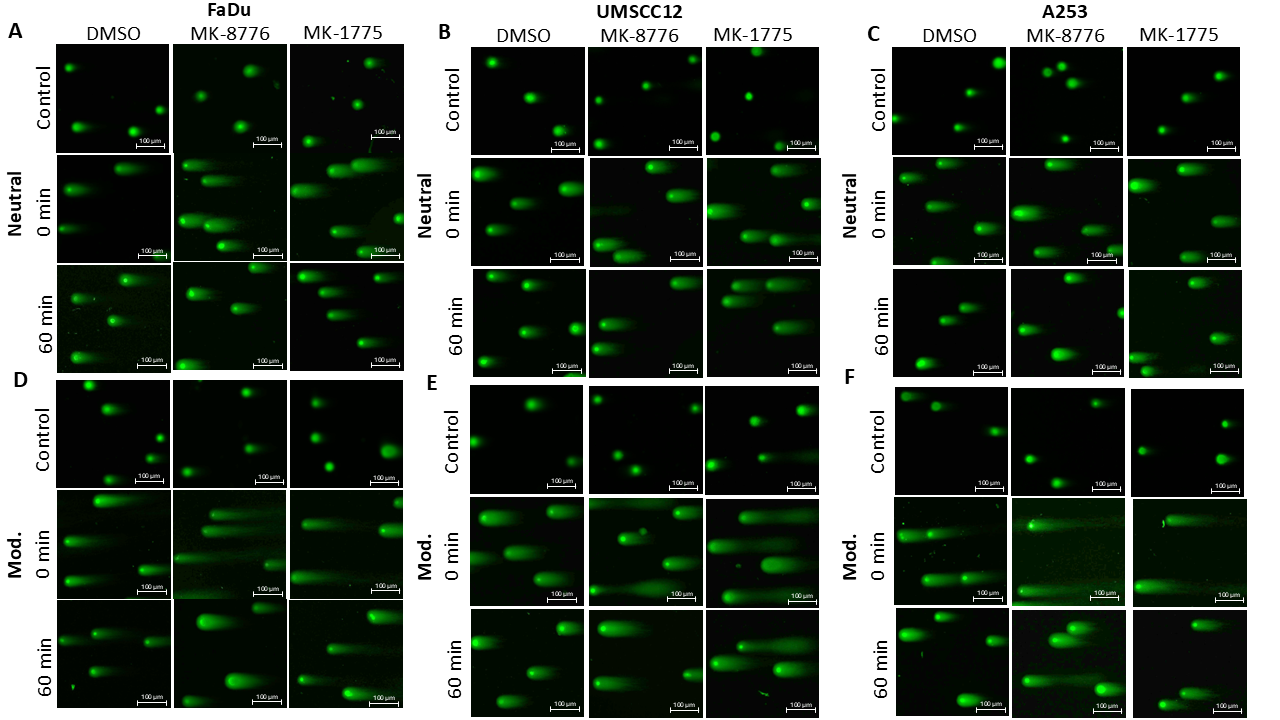


**Supplementary Figure 22. Chk1 or Wee1 inhibition causes increased persistence of high-LET PBT-induced DSBs.** (A,D) FaDu, (B,E) UMSCC12 or (C,F) A253 cells were treated with 1 μM MK-8776 (10 μM for FaDu) or 0.2 μM MK-1775 for ~16 h prior to exposure to 4 Gy high-LET PBT. (A-C) DSBs and (D-F) CDD were measured at various timepoints post-irradiation using the enzyme-modified neutral comet assay. Shown are representative images for the unirradiated controls, 0 min and 60 min post-irradiation. Scale bar is 100 μm.


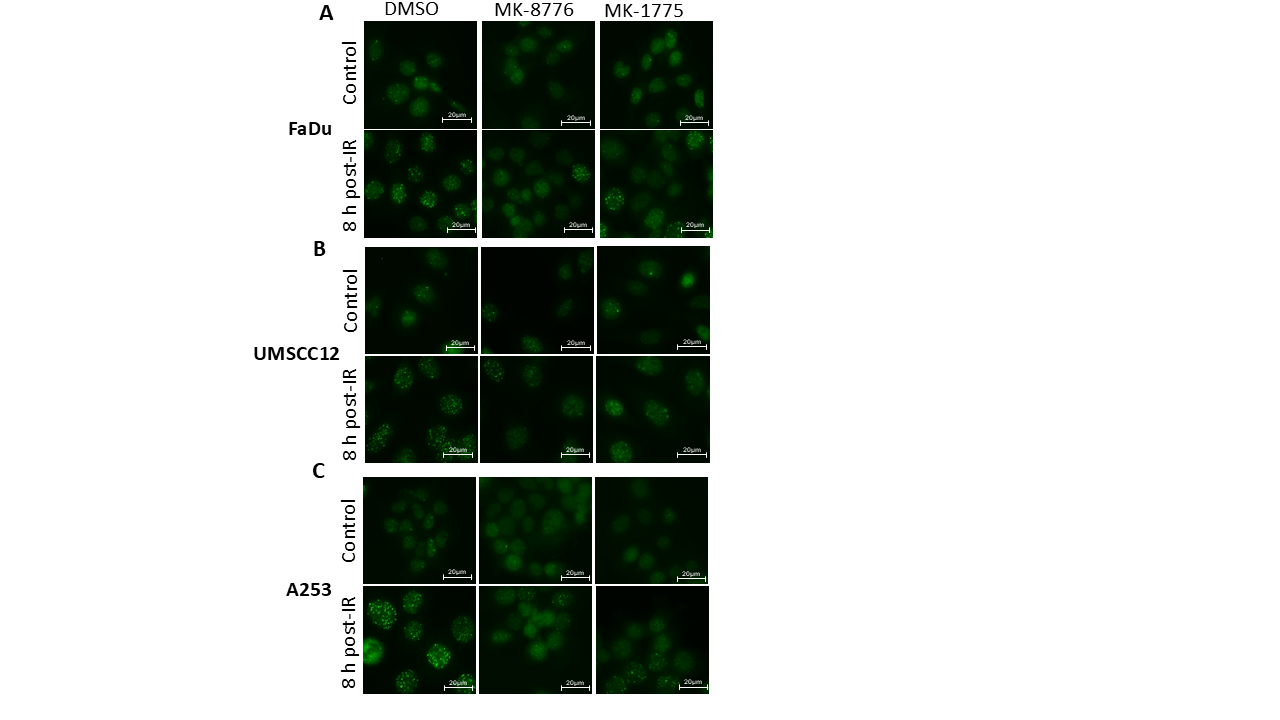


**Supplementary Figure 23. Chk1 or Wee1 inhibition causes increased persistence of high-LET PBT-induced DSBs.** (A) FaDu, (B) UMSCC12 or (C) A253 cells were treated with 1 μM MK-8776 (10 μM for FaDu) or 0.2 μM MK-1775 for ~16 h prior to exposure to 4 Gy high-LET PBT. DNA DSB repair was measured at various timepoints post-irradiation using RAD51 foci through immunofluorescence microscopy. Shown are representative images for the unirradiated controls, plus 8 h post-irradiation. Scale bar is 20 μm.


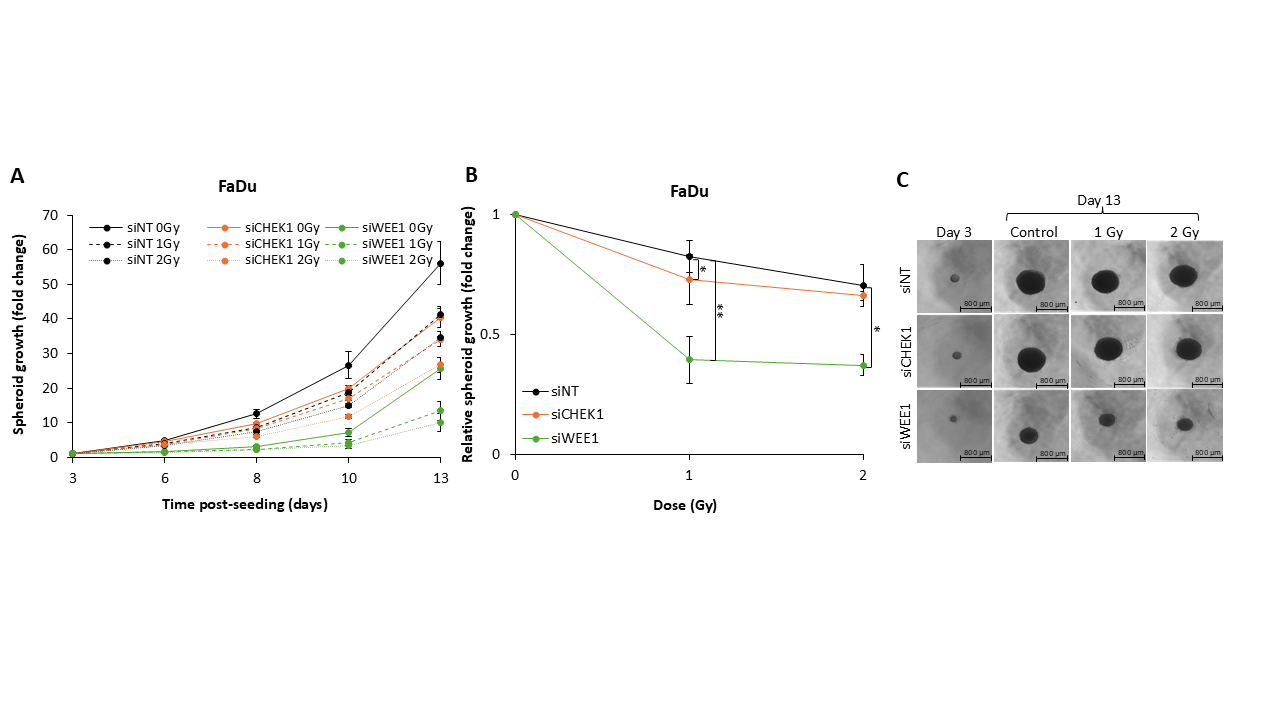


**Supplementary Figure 24. siRNA knockdown of CHEK1 or WEE1 radiosensitises FaDu spheroids to X-ray radiation.** FaDu cells were treated with siRNA targeting either CHEK1 (siCHEK1), WEE1 (siWEE1) or a non-targeting siRNA (siNT) for 48 h, and then spheroids allowed to form in ultra-low attachment plates. (A) Spheroids were irradiated with X-rays (1 or 2 Gy) on day 3 and volumetric growth was monitored for a further 10 days. Shown is the mean fold change in volume±SE. (B) Fold change in spheroid volume from days 3-13 was calculated as a function of radiation dose, and data normalised to the unirradiated controls which was set to 1.0. *p<0.05, **p<0.02 as analysed by a one sample *t*-test. (C) Representative images of spheroids on days 3 or 13 are shown for each condition. Scale bar is 800 μm.


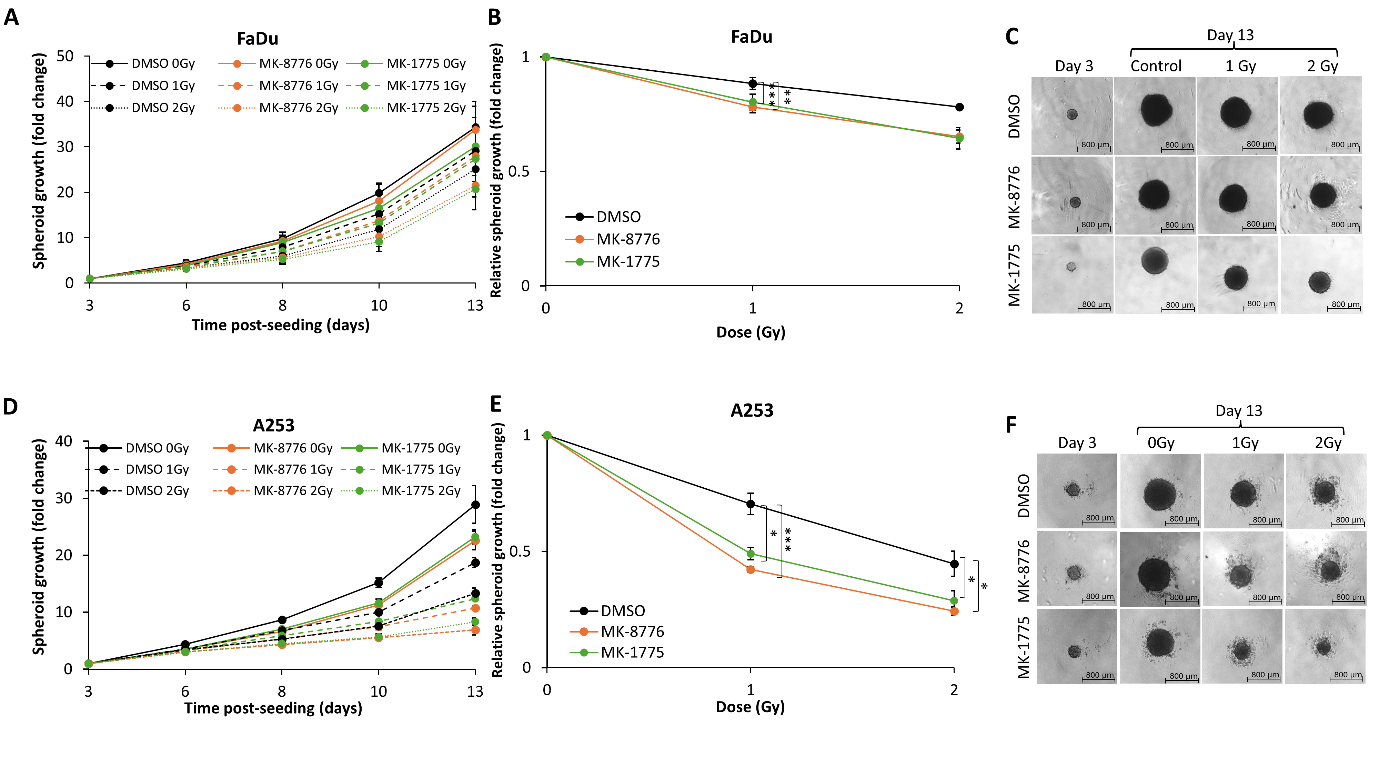


**Supplementary Figure 25. Chk1 or Wee1 inhibition radiosensitises HNSCC spheroids to X-ray radiation.** (A-C) FaDu or (D-F) A253 cells were seeded in ultra-low attachment plates for 48 h to allow spheroids to form, and then treated with either 0.5 μM MK-8776 or 0.1 μM MK-1775 for 1 h prior to exposure to X-ray radiation (1 or 2 Gy). (A, D) Volumetric growth of the spheroids was monitored for a further 10 days post-irradiation and shown is the mean fold change in volume±SE. (B, E) Fold change in spheroid volume from days 3-13 was calculated as a function of radiation dose, and data normalised to the unirradiated controls which was set to 1.0. *p<0.05, **p<0.02, ***p<0.002 as analysed by a one sample *t*-test. (C, F) Representative images of spheroids on days 3 or 13 are shown for each condition. Scale bar is 800 μm.


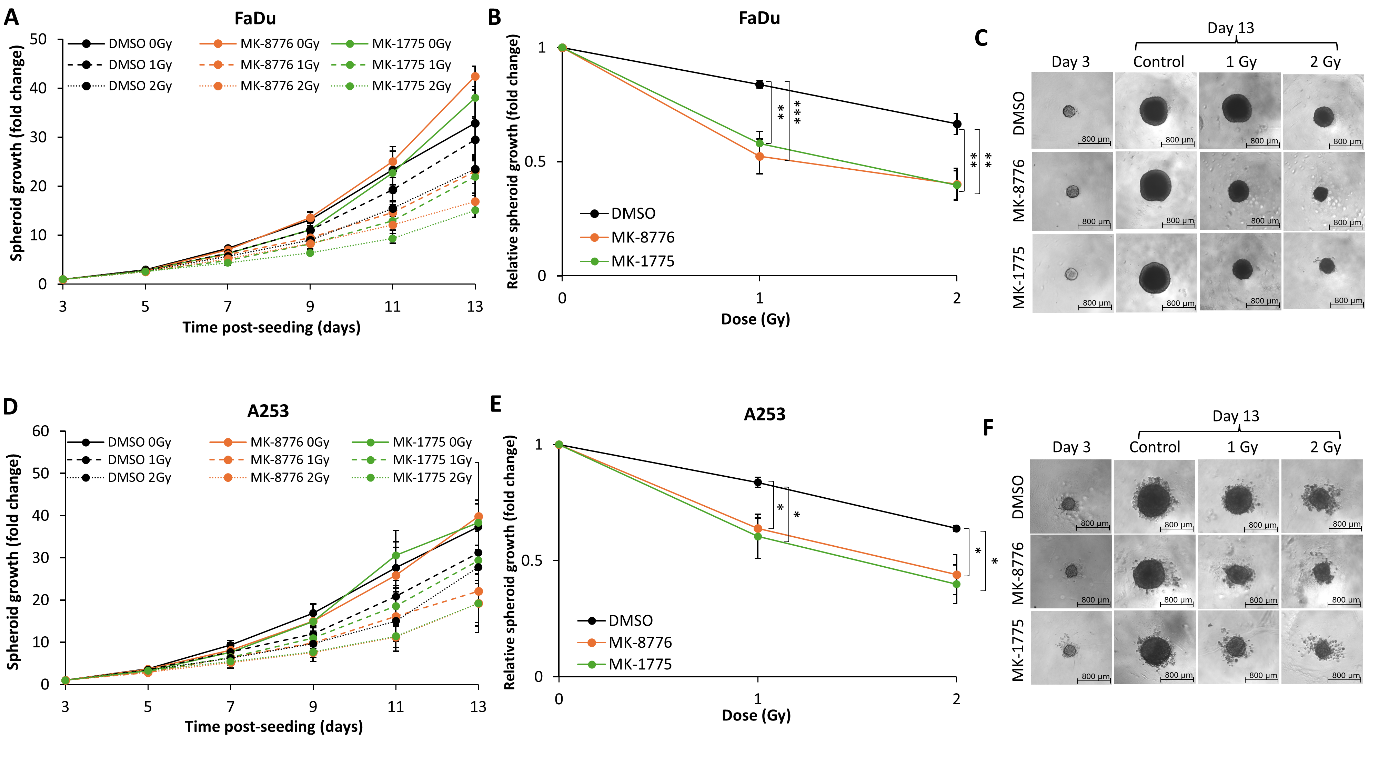


**Supplementary Figure 26. Chk1 or Wee1 inhibition radiosensitises HNSCC spheroids to low-LET PBT.** (A-C) FaDu or (D-F) A253 cells were seeded in ultra-low attachment plates for 48 h to allow spheroids to form, and then treated with either 0.5 μM MK-8776 or 0.1 μM MK-1775 for 1 h prior to exposure to low-LET PBT (1 or 2 Gy). (A, D) Volumetric growth of the spheroids was monitored for a further 10 days post-irradiation and shown is the mean fold change in volume±SE. (B, E) Fold change in spheroid volume from days 3-13 was calculated as a function of radiation dose, and data normalised to the unirradiated controls which was set to 1.0. *p<0.05, **p<0.01, as analysed by a one sample *t*-test. (C, F) Representative images of spheroids on days 3 or 13 are shown for each condition. Scale bar is 800 μm.

**Supplementary Table 1.** Dose enhancement ratios (DER) at 50 % survival for HNSCC cell lines following siRNA knockdown of CHEK1 or WEE1 with X-ray radiation.

| **DER_50%_** | **Cell Line** | |
| --- | --- | --- |
| Treatment | **FaDu** | **UMSCC12** |
| siCHEK1 | 1.53 | 1.44 |
| siWEE1 | 1.46 | 1.37 |
